# Metabolite Profiling of Malaysian *Gracilaria edulis* Reveals Eplerenone as Novel Antibacterial Compound for Drug Repurposing Against MDR Bacteria

**DOI:** 10.3389/fmicb.2021.653562

**Published:** 2021-06-30

**Authors:** Ali Asghar, Yong-Chiang Tan, Muhammad Shahid, Yoon-Yen Yow, Chandrajit Lahiri

**Affiliations:** ^1^Department of Biological Sciences, Sunway University, Petaling Jaya, Malaysia; ^2^Department of Food Sciences, Universiti Kebangsaan, Bangi, Malaysia

**Keywords:** antimicrobial resistance, *Gracilaria edulis*, red seaweed, bioactive compounds, virtual screening, eplerenone

## Abstract

With a continuous threat of antimicrobial resistance on human health worldwide, efforts for new alternatives are ongoing for the management of bacterial infectious diseases. Natural products of land and sea, being conceived to be having fewer side effects, pose themselves as a welcome relief. In this respect, we have taken a scaffolded approach to unearthing the almost unexplored chemical constituents of Malaysian red seaweed, *Gracilaria edulis*. Essentially, a preliminary evaluation of the ethyl acetate and acetone solvent extracts, among a series of six such, revealed potential antibacterial activity against six MDR species namely, *Klebsiella pneumoniae*, *Pseudomonas aeruginosa*, *Salmonella enterica*, methicillin-resistant *Staphylococcus aureus* (MRSA), *Streptococcus pyogenes*, and *Bacillus subtilis*. Detailed analyses of the inlying chemical constituents, through LC-MS and GC-MS chromatographic separation, revealed a library of metabolic compounds. These were led for further virtual screening against selected key role playing proteins in the virulence of the aforesaid bacteria. To this end, detailed predictive pharmacological analyses added up to reinforce Eplerenone as a natural alternative from the plethora of plausible bioactives. Our work adds the ongoing effort to re-discover and repurpose biochemical compounds to combat the antimicrobial resistance offered by the Gram-positive and the -negative bacterial species.

## Introduction

Antimicrobial resistance (AMR) has become a critical concern for public health worldwide. Numerous pathogens have started to evolve and progressively develop resistance toward currently in-practice antibiotics, thereby adding, in essence, to global morbidity and mortality for the diseases caused by these pathogens (Subramani et al., 2017). Besides such evolutionary constraints, multidrug resistance (MDR) in bacterial strains, also spreads from irrational usages of antibiotics (Shrestha et al., 2018). In this regard, several pathogens have posed resistance toward different types of antibiotics. Notable among them is vancomycin-resistant *Enterococcus faecium*, methicillin-resistant *Staphylococcus aureus* (MRSA), carbapenem-resistant *Acinetobacter baumannii* and *Pseudomonas aeruginosa*, clarithromycin-resistant *Helicobacter pylori*, fluoroquinolone-resistant *Salmonella* and *Campylobacter* spp., cephalosporin- and fluoroquinolone-resistant *Neisseria gonorrhoeae* and carbapenem-resistant and extended-spectrum beta-lactamase (ESBL) creating *Enterobacteriaceae* (World Health Organization [WHO], 2017). Moreover, the development of new antibiotics has slowed down over the past few decades while the emergence rate of resistant pathogens is high (Ventola, 2015). This necessitates an immediate exploration of novel antibacterials to combat the MDR threats.

To unearth novel antimicrobials for clinicians and pharmacological industries, efforts are focused on exploring the natural products from land and sea. This is due to the fact that natural products, with effective antimicrobial potency, have been proven successful in sufficiently reducing the worldwide load of infectious illnesses (Mérillon and Rivière, 2018). They are, therefore, used as alternative agents to eradicate the extensive use of synthetic drugs and related antibiotics (Cheesman et al., 2017). In fact, marine organisms, such as seaweeds and diatoms, are considered as novel sources of bioactive constituents which produce a vast diversity of secondary metabolites, revealing a broad spectrum of biological actions (El Shafay et al., 2016). In this context, Ismail et al. (2016) have screened different types of seaweeds, namely, green *Cladophora pellucida*, brown *Padina tetrastromatica*, and red *Laurencia papillosa* for the isolation of antimicrobial agents.

In line with the exploration of seaweeds for novel antimicrobials, secondary metabolites like phlorotannins, acrylic acid, terpenoids, phenolic compounds, steroids, halogenated ketones, alkaline, fatty acid and cyclic polysulphides have been unearthed (Cheung et al., 2015; Khelil-Radji et al., 2017). These bioactive constituents have biological potentials including dietary, antifungal, anti-inflammatory, anti-ageing, antibacterial, antioxidant, anticancer, anticoagulation, anti-malarial and anti-proliferation (Chan et al., 2015; Agregán et al., 2017). Moreover, growing evidence has revealed that red seaweeds contain a broad spectrum of secondary metabolites with antibacterial potentials (Kasanah et al., 2019). In fact, in Asian countries, red seaweeds are generally used as food materials and find extensive usages as seasonings, sushi wrappings, condiments, vegetables, noodles, and for polysaccharide production for the food and pharmaceutical industries (Seedevi et al., 2017). In this regard, the red seaweed, *Gracilaria edulis*, has attracted extensive consideration due to its biological and pharmacological applications and several therapeutic benefits.

With a broad range of biological activities of *G. edulis*, encompassing antibacterial, anticoagulant, antidiabetic, antioxidant, antiproliferative and anti-inflammatory activities (De Almeida et al., 2011), they have been considered as likely natural functional foods (Rosemary et al., 2019). In fact, few researchers have even reported its *in vitro* antibacterial activities. For instance, Hemasudha et al. (2019) determined the antibacterial activity of the methanol extract of *G. edulis* against *S. aureus* and *P. aeruginosa*, having a significant activity, with inhibition zone of 18mm and 19mm being recorded, respectively. Similarly, Umakanthan et al. (2017) described the antibacterial effects of the crude extract of *G. edulis* against *S. aureus* and *Escherichia coli*. Moreover, Kasanah et al. (2019) reported the antibacterial activity against bacterial fish pathogens like *Vibrio fluvialis* and *Vibrio compbelii*. Again, Arulkumar et al. (2018) confirmed the methanol extract of *G. edulis* to have remarkable inhibitory activity toward *Bacillus subtilis*, *S. aureus*, *E. coli* and *Pseudomonas fluorescens*.

In this study, thus, we have detailed a stepwise approach of determining the efficacy of different solvent-based crude extracts of Malaysian *G. edulis* against clinically important MDR bacterial species namely, *Klebsiella pneumoniae*, *P. aeruginosa*, *Salmonella enterica*, MRSA, *Streptococcus pyogenes* and *B. subtilis*. Further chemical profiling of the extracts through LC-MS and GC-MS revealed their potential chemical determinants. These were screened virtually and pharmacologically to unveil potential novel bioactive compounds. To this end, a molecular dynamics simulation by our shortlisted bioactive compounds, Eplerenone, on selected virulent protein targets of the aforementioned bacteria, revealed its high potential as an effective antibacterial compound, targeting both the gram-positive and the -negative pathogens.

## Materials and Methods

### Collection of Materials

#### Solvents

For crude extractions, HPLC grade organic solvents with increasing polarities were used. These comprised chloroform (99.9%, Sigma-Aldrich, LiChrosolv, Malaysia), ethyl acetate, acetone (99.5% Chemiz, Malaysia), ethanol, methanol (99.8%, ChemAR, Systerm, Malaysia) and double-distilled Milli-Q Type 1 water (MilliporeMerck, Germany). For LC-MS and GC-MS analyses, however, MS grade solvents were used.

#### Seaweeds

Healthy specimens of *Gracilaria edulis* were collected from Pantai Morib, Selangor, Malaysia.

#### Bacterial Strains

A total of six clinical isolates, including three each of Gram-positive and Gram-negative, were used in the study. These comprised *Bacillus subtilis* (ATCC-11774), *Streptococcus pyogenes* (ATCC-49399), methicillin-resistant *Staphylococcus aureus* (MRSA) (MTCC-381123), *Klebsiella pneumoniae* (ATCC-700603), *Pseudomonas aeruginosa* (ATCC-10145), and *Salmonella enterica* (ATCC-14028), these strains were received from the Department of Biological Sciences (DBS), School of Medical and Life Sciences, Sunway University, Malaysia. All these six bacterial strains were found to be resistant to at least five of the ten antibiotics tested for their resistivity/sensitivity profile and thus, considered to be multidrug resistant (Supplementary Table 1).

### Crude Extracts Preparation

Extracts of *G. edulis* were prepared through two different approaches, namely, sequential and direct, following the procedure of Subermaniam et al. (2020). For the sequential process, the aforementioned solvents were used in the order of increasing polarity *viz.* chloroform < ethyl acetate < acetone < ethanol < methanol < water. For the direct extracts, ethyl acetate and acetone were used only. Essentially, for both approaches, the seaweeds were rinsed sequentially with seawater followed by normal tap and then double distilled water to eradicate dirt and impurities. Clean samples were then dried using a freeze-dryer and later crushed into fine granule powder using an electric grinder. Different fractions of extracts were prepared using 10 grams of each powder to dissolve them in 100 mL of the above-mentioned solvents. All the prepared mixtures were made homogeneous using a rotating shaker (Yihder LM-530D, Shaker, Taiwan) for 24 h and finally centrifuged (Eppendorf 5810 R Centrifuge, Germany) at 4000 rpm for 10 min at 4°C to separate the supernatant. Each of the clear supernatants of the extracts was concentrated via a Rotary evaporator (Thermo Fisher Scientific EYELA N-1200A Rotary Evaporator, Tokyo). A further concentration using a vacuum concentrator (LaboGene, Brigachtal, Germany) was done to obtain a viscous liquid for storage at 4°C and future experiments.

### Potential *in vitro* Antibacterial Activities

#### Disc-Diffusion Test

All the microorganisms were tested for their response against the crude extract fractions (CEF) of *G. edulis*. Essentially, a loopful of inocula was swabbed on plates containing Mueller-Hinton Agar (MHA, Oxoid). This was followed by soaking 6 mm sterilized paper discs with different crude extract fractions and placing them on those plates to be incubated for 16 h at 37°C. Gentamicin having a concentration of 10 μg/disc served as a positive control along with DMSO (<1%) as solvent control. The activities of each of the CEF were recorded by measurement of the diameter of inhibition zones. All experiments were performed with technical triplicates, and twice, to render two biological duplicates.

#### Broth Dilution Assay

To assess the minimum inhibitory concentration (MIC) values of the CEF, broth dilution assay was used, following Clinical & Laboratory Standards Institute (CLSI) measures. This was initiated by adding 5 μL of each CEF onto 96-well plates, containing 5 × 10^5^ CFU/mL bacterial cells, to a range of final concentrations from 250 to 2000 μg/mL. These plates were incubated for 16 h at 37°C. Three different controls were maintained for each trial. These were Gentamicin (10 μg/mL) as the positive control, DMSO < 1% as solvent control and bacterial inoculum as the negative control. The MIC_50_ value was determined from the lowest concentration of the tested CEF, displaying inhibitory result toward the pathogens, as recorded through the Microplate reader (TECAN, Infinite-M200-PRO). Biological duplicates were used to confirm each trial having technical triplicates. The CEF of ethyl acetate (EA) and acetone (AC), having notable results, were utilized for further chromatographic separation analyses.

### Statistical Tests

All the tests in the present study were performed in triplicates and the data obtained are expressed as the mean ± standard deviation (S.D). The *P*-values were determined using student’s *T*-test, two-tailed distribution, where ^∗^ refers to *P* ≤ 0.05. These have been reflected in Tables 1, 2, Supplementary Figure 1, and Supplementary Tables 4, 5.

**TABLE 1:** Antibacterial activity of *G. edulis* rhizome sequential and direct crude extracts via disc diffusion.

Zones of Inhibition (mm)						
Bacteria	P.C	S.C	EA	AC	EA(D)	AC(D)
*B. subtilis*	32.00 ± 0.70	−	−	−	9.26 ± 0.52	7.00 ± 0.56
MRSA		−	−	−	−	−
*S. pyogenes*		−	−	−	−	−
*P. aeruginosa*		−	−	−	−	−
*K. pneumoniae*		−	−	−	6.5 ± 0.42	6.8 ± 0.14
*S. enterica*		−	−	−	−	−

*“−” no activity, P.C: positive control (Gentamicin 10 μg), SC: solvent control (DMSO < 1%), EA: ethyl acetate, AC: acetone, EA(D): ethyl acetate direct; AC(D): acetone direct. The data is expressed as the mean ± standard error of two independent experiments performed in technical triplicates.*

**TABLE 2:** MIC_50_ of the *G. edulis* sequential and direct extracts against all tested pathogens.

MIC_50_ (μ g/mL)				
Microorganisms	EA	AC	EA(D)	AC(D)
*B. subtilis*	2000	2000	250	250
MRSA	ND	ND	250	250
*S. pyogenes*	ND	ND	250	250
*P. aeruginosa*	ND	2000	250	250
*K. pneumonia*	ND	ND	250	250
*S. enterica*	2000	ND	1000	250

*ND: not determined, AC: acetone, ET: ethanol, EA(D): ethyl acetate direct; AC(D): acetone direct. The illustrated MIC_50_ values are the lowest inhibitory concentrations achieved from two independent trials performed in triplicate.*

### Exploration of Chemical Constituents Through Chromatographic Analyses

#### Liquid Chromatography With Mass Spectrometry (LC-MS)

To gain a detailed understanding of the standard and novel chemical constituents of *G*. *edulis*, the EA and AC CEF were analyzed via LC-MS as per the method described by Yap et al. (2019). Systematic errors were eliminated by using reference solution along with the two ions, having m/z of 121.0508 and 92266.0097, being selected for mass calibration. To this end, the mass spectra for the chemical constituents for the said CEF were run against the database of NIST (National Institutes of Standard and Technology, Gaithersburg, MD, United States) via the Mass Spectral Search Program-2009 version 2, for the documentation of homologous chemical compounds through the Agilent Mass-Hunter Qualitative Analysis B.05.00 software.

### Gas Chromatography With Mass Spectrometry (GC-MS)

To gain a further understanding of the constituents in the CEF of the volatile solvents EA and AC, they were subjected to gas chromatographic followed by mass spectrometric analysis, using Agilent technologies model 7890B GC System coupled with Pegasus HT High Throughput TOFMS (Leco Corp., MI, The United States). Typically, an aliquot of an extract of 1ml was injected into the GC-MS apparatus. To this end, Agilent J&W HP-5MS analytic column comprising phenyl methyl siloxane and having the dimensions of length 30 m, Dia. 0.32 mm, Film, 0.25 μm, was used to separate components under an inert atmosphere of helium (1.5 mL/min). Additional standardized parameters utilized during the process comprised an initial oven temperature of 80°C (2 min) increased to 300°C at the rate of 3°C/min, a solvent delay time of 5 min, an inlet line temperature of 225°C, along with an ion source temperature of 250°C. Keeping a GC run time of sixty-four (64) mins, the mass spectra readings were taken at 70 eV along with an acquisition mode-scan of 20-1000 amu. Using a database of NIST libraries, these mass spectral results were interpreted and deciphered phytochemicals were documented.

### *In silico* Analysis of Chemical Determinants on Selected Protein Targets

#### Database Searching and Model Acquisition

An estimation of the efficacies of the chemical determinants as antibacterials were obtained through their *in silico* analyses against target proteins of the six bacteria namely, *S. enterica*, *K. pneumoniae*, *P. aeruginosa*, Methicillin-Resistant *S. aureus*, *S. pyogenes*, and *B. subtilis*. In this regard, all virulence factors (VFs), occurring in the proteome of these bacteria were identified through the Virulence Factor Database (VFDB) (Liu et al., 2019). These VFs were being queried on Protein Data Bank (PDB) (Berman et al., 2000) to search for the available crystallized 3D structures of the proteins. Of these, two were selected from each bacteria of interest based on their physiological importance, the resolution, and sequence coverage for subsequent analyses, prioritizing the full-length crystallized proteins (Supplementary Table 2). Essentially, PrgK and PrgH for *S. enterica*, IucA and IucC for *K. pneumoniae*, WaaP and AlgE for *P. aeruginosa*, CapE and EsxA for *S. aureus*, LepA and SmeZ-2 for *S. pyogenes* as well as DhbE and CesB for *B. subtilis*, were selected. Moreover, validated homology models of the DnaK protein from *P. aeruginosa* and *S. aureus* were retrieved from a previous study (Asghar et al., 2020).

#### Prediction of Druggable Pockets

**FIGURE 1:**
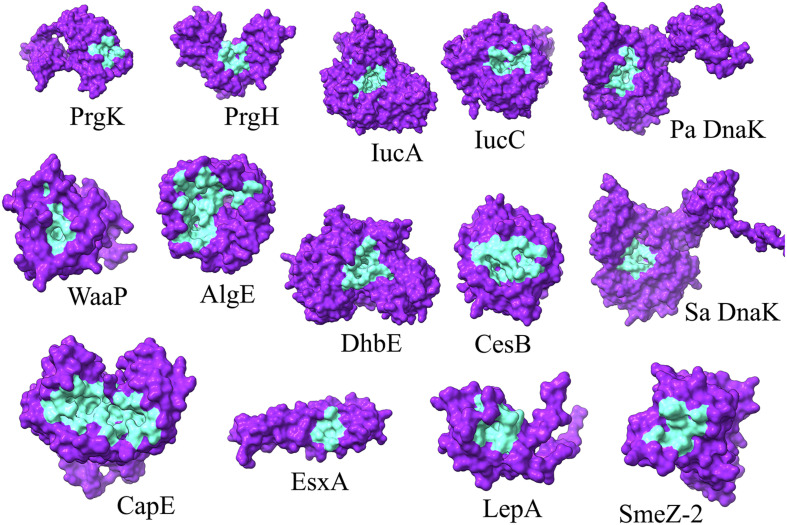
3D representation of the P2Rank predicted druggable pockets (cyan) of selected proteins (indigo). Pa and Sa denote *P. aeruginosa* and *S. aureus*, respectively.

To identify the druggable pockets of each selected target protein for further structure-based screening, the 3D localization of the druggable pockets were predicted via a template-independent machine learning-based approach known as P2Rank (Krivák and Hoksza, 2018). The top-ranked predicted pockets, for each protein, were carried over for further analyses (Figure 1 and Supplementary Table 2).

#### Structure-Based Virtual Screening of Chemical Compounds

The ability of each chemical compounds identified from prior chromatographic extractions to target the selected target proteins at the predicted druggable pockets were assessed via POAP, an *in silico* virtual screening pipeline powered by AutoDock 4.2 (Morris et al., 2009; Samdani and Vetrivel, 2018). Before the preparation of the ligands, those consisting of uncommon atoms, such as Silicon, Nickel, and Molybdenum, were excluded from further analyses to avoid inaccuracies. Moreover, ligands with more than 32 torsions were excluded as well as per AutoDock 4.2’s limitation. Following the POAP Ligand Preparation pipeline, SMILES notations of the ligands were converted into 3D structures. The MMFF94 force field was utilized for ligand optimization before virtual screening. Moreover, the best conformers were selected for each ligand from the 50 conformers generated through Weighted Rotor Search. Furthermore, the ligands were minimized via the conjugate gradient algorithm for 5000 steps. Before PDBQT file generation, physiological protonation states of the ligands were generated using the Chimera addH function (Pettersen et al., 2004).

For the target proteins, the PDB retrieved structures were cleaned before their PDBQT structures were generated, of which non-amino acid residues were removed and structural optimizations were carried out via the Chimera Dock Prep function (Pettersen et al., 2004). The grids, for virtual screening space of search, were set to encompass the docking pockets previously predicted by P2Rank (Supplementary Table 3).

#### Pharmacological Properties Screening

The chemical compounds were screened for drug-likeness rules and ADME properties via SwissADME (Daina et al., 2017). Essentially, the potentially druggable molecules were screened through pharmacological properties encompassing the properties of absorption, good gastrointestinal (GI) absorption, bad BBB permeability and non-P-glycoprotein (PGP) substrates. Moreover, concerning metabolism, we prioritize non-cytochrome P450 inhibitors to avoid adverse effects upon the co-administration of drugs. Furthermore, we have considered five rules for ascertaining the drug-likeness, namely, Lipinski, Ghose, Veber, Egan, and Muegge rules (Ghose et al., 1999; Egan et al., 2000; Muegge et al., 2001; Veber et al., 2002; Lipinski, 2004). Finally, higher bioavailability scores (Abbot Bioavailability Score) were preferred to ensure high drug efficiency (more than 10%) upon oral administration (Hann and Keseru, 2012).

## Results

### Variable Antibacterial Activity of Crude Extracts by Disc Diffusion

Disc diffusion technique was utilized to perform an initial screening for the antibacterial activities of the *G. edulis* different solvent-based CEF, prepared freshly, for all the trials. In each case, the solvent control (SC), DMSO, displayed no inhibition zone thereby establishing no antibacterial activity toward the tested bacterial species (TBS). Furthermore, none of the sequential CEF revealed activity toward the TBS (Table 1 and Supplementary Table 4), although, in the case of direct CEF, the scenario was fairly different. Extract EA(D) and AC(D) showed moderate ZOI toward *B. subtilis* and *P. aeruginosa* but did not show activity against the rest of the TBS (RTBS) (Table 1).

### Antibacterial Screening of Crude Extracts via Broth Dilution

A broth dilution test was used for estimating the antibacterial activity of *G. edulis* crude extracts by calculating the inhibition percentage of each TBS. This, in turn, demonstrated the percentages of antibacterial effect of EA and AC, toward the TBS, at concentrations of 250 and 2000 μg/mL, respectively for direct and sequential CEF. In the case of sequential fraction, the extract from EA revealed the maximum percentage (60) of antibacterial activity against *B. subtilis* followed by *S. enterica* (50) while that from AC exhibited 50% effect toward *B. subtilis* and 60% against *K. pneumoniae*. No positive results were detected either against the RTBS (Supplementary Figures 1a-f) or for the other solvent CEF of chloroform (CF), ethanol (ET), methanol (MT), and aqueous (water, WT) toward all TBS (Supplementary Table 5). In direct extracts, CEF of EA displayed inhibition of 90% against *B. subtilis* along with 80, 70, 78, 75, and 72% for MRSA, *S. pyogenes*, *P. aeruginosa, K. pneumonia* and *S. enterica*, respectively. Remarkably, all TBS were inhibited by AC CEF and the percentage of antibacterial effects were 85, 82, 85, 89, 80, and 80, respectively, for *B. subtilis*, MRSA, *S. pyogenes*, *P. aeruginosa, K. pneumonia* and *S. enterica* (Supplementary Figures 1g-l). The MIC_50_ value of all the extracts are presented in Table 2.

The results obtained from the *G. edulis* CEF of EA and AC offered prominent antibacterial efficacy. This enabled us to use the CEF to further process them for the identification of bioactive compounds through LC-MS and GC-MS analyses.

### Exploration of Chemical Components Through Liquid Chromatography–Mass Spectrometry (LC-MS) Analysis

For a fast, mass-directed investigation of the probable chemical components of *G. edulis*, its EA and AC sequential CEF were exposed to LC-MS analysis (Supplementary Figures 2, 3). The compounds were matched with the identity of known molecules on the Metlin database, a threshold Molecular Formula Generator (MFG) with scores above 86% along with a ± 2% difference. This revealed 22 and 29 matched (Supplementary Tables 6, 7) and 40 and 32 unmatched compounds, for EA and AC CEF, respectively (Supplementary Tables 8, 9). The 51 matched compounds, above the MFG threshold value, were further explored for their reported antibacterial activities and carried forward for virtual screening. Notably, out of a total of 51, 13 compounds from both groups of EA and AC CEF were redundant and the rest 31 have not been reported to date with any antibacterial activities (Supplementary Tables 6, 7).

### Identification of Volatile Constituents by Gas Chromatography–Mass Spectrometry (GC-MS)

To find any volatile organic bioactive compounds, existing in the EA and AC sequential & direct CEF of Malaysian *G. edulis*, they were subjected to GC-MS analysis (Supplementary Figures 4-7). The chromatogram of the compounds presented mentionable area% scores (above 0.5%). This revealed 12 and 8 compounds, for the sequential EA and AC CEF, respectively (Table 3) along with 15 and 7 for the direct CEF of EA & AC, respectively (Table 4). Combining the total number of 42 compounds obtained from the GC-MS analyses of EA and AC sequential & direct CEF and removing the redundant ones, the unique number appears to be 28 out of which 25 compounds revealed to have not been associated with any antibacterial activities (Tables 3, 4).

**TABLE 3:** Compounds existing in *G. edulis* ethyl acetate and acetone (S) extract identified by GC-MS analysis.

No	Extracts	Identified compounds	Molecular formula	R.T. (Min)	Area%	Antibacterial activity report
1	**EA(S)**	3,8,13,18-Tetraethyl-2,7,12,17-tetramethyl-7, 8-(diacetyl) methylene-7,8-dihydro-21H,23H-porphine copper (II)	C_37_H_42_CuN_4_O_2_	32.09	4.035	NR
2		Phenol, 2,4-bis(1,1-dimethylethyl)	C_14_H_22_O	14.41	3.925	Reported (Padmavathi *et al.*, 2014)
3		Phytol	C_20_H_40_O	26.67	2.3	Reported (Ghaneian *et al.*, 2015)
4		Decanoic acid, ethyl ester	C_12_H_24_O_2_	24.54	1.787	NR
5		n-Hexadecanoic acid	C_16_H_32_O_2_	23.98	5.008	NR
6		3-Methyl-1,2-diazirine	C_2_H_4_N_2_	3.01	7.529	NR
7		1-Octadecyne	C_18_H_34_	21.52	2.2	NR
8		Diisooctyl phthalate	C_24_H_38_O_4_	33.77	0.914	NR
9		1,2-Benzenediol bis(trimethylsilyl) ether	C_12_H_22_O_2_Si_2_	57.93	2.052	NR
10		Nickel tetracarbonyl	C_4_NiO_4_	3.1	1.797	NR
11		4-Penten-2-one, 4-methyl-	C_6_H_10_O	10.91	1.628	NR
12		Molybdenum, bis[(1,2,3,4,5-ü)-1,3-bis(1,1-dimethylethyl)-2,4-cyclopentadien-1-yl] di-ae- carbonyldicarbonyldi-, (mo-mo)	C_16_H_10_Mo_2_O_6_^–6^	52.89	0.646	NR
1	**AC(S)**	Phenol, 2,4-bis(1,1-dimethylethyl)-	C_14_H_22_O	14.41	0.771	Reported (Padmavathi *et al.*, 2014)
2		n-Hexadecanoic acid	C_16_H_32_O_2_	24.08	9.078	NR
3		Diisooctyl phthalate	C_24_H_38_O_4_	33.77	3.878	NR
4		26-Nor-5-cholesten-3á-ol-25-one	C_26_H_42_O_2_	45.57	1.725	NR
5		Hexadecanoic acid, ethyl ester	C_18_H_36_O_2_	24.54	2.074	NR
6		Arachidonic acid	C_20_H_32_O_2_	30.23	1.039	Reported (Beavers *et al.*, 2019)
7		Nickel tetracarbonyl	C_4_NiO_4_	7.51	1.329	NR
8		2-Heptyl-1,3-dioxolane	C_10_H_20_O_2_	53.21	2.882	NR

*NR, Not reported; S, sequential extracts.*

**TABLE 4:** Compounds existing in *G. edulis* ethyl acetate and acetone (D) extracts identified by GC-MS analysis.

No	Extracts	Identified compounds	Molecular formula	R.T. (Min)	Area%	Antibacterial activity reports
1	**EA(D)**	Phenol, 2-methoxy-3-(2-propenyl)-	C_10_H_12_O_2_	10.73283	6.3257	NR
2		Phenol, 2,4-bis(1,1-dimethylethyl)	C_14_H_22_O	14.44737	7.7157	Reported (Padmavathi et al., 2014)
3		n-Hexadecanoic acid	C_16_H_32_O_2_	24.12967	9.1086	NR
4		Diisooctyl phthalate	C_24_H_38_O_4_	33.80467	0.56258	NR
5		Cholesterol	C_27_H_46_O	45.68133	32.831	NR
6		Non-adecane	C_19_H_40_	18.65483	4.034	NR
7		Decanoic acid, ethyl ester	C_12_H_24_O_2_	24.5795	5.3174	NR
8		Arachidonic acid	C_20_H_32_O_2_	30.26833	1.4028	Reported (Beavers et al., 2019)
9		1-Octadecyne	C_18_H_34_	21.55767	0.63897	NR
10		Silane, tetramethyl	C_4_H_12_Si	61.334	0.18473	NR
11		2-Tridecanone	C_13_H_26_O	21.6535	0.66248	NR
12		Oxalic acid, cyclobutyl octadecyl ester	C_24_H_44_O_4_	28.1485	0.15812	NR
13		Ribo-ribo disaccharide	C_10_H_18_O_9_	59.13117	0.59025	NR
14		[2,2′-Bifuran]-3-carboxylic acid, 5′- methyl-, methyl ester	C_11_H_10_O_4_	14.44403	7.7157	NR
15		á Carotene	C_40_H_56_	27.802	0.9908	NR
1	**AC(D)**	Phenol, 2-methoxy-3-(2-propenyl)-	C_10_H_12_O_2_	10.73117	8.9936	NR
2		Decanoic acid, ethyl ester	C_12_H_24_O_2_	24.57283	2.2412	NR
3		n-Hexadecanoic acid	C_16_H_32_O_2_	23.96033	1.2408	NR
4		Diisooctyl phthalate	C_24_H_38_O_4_	33.79967	0.85802	NR
5		Cholest-5-en-3-ol	C_27_H_48_O	45.63817	3.4662	NR
6		Ar-tumerone	C_15_H_20_O	17.86533	1.7063	Reported (Marliyana et al., 2019)
7		Phenol, 2,4-bis(1,1-dimethylethyl)-	C_14_H_22_O	14.44403	4.6668	Reported (Padmavathi et al., 2014)

*NR, Not reported; D, direct extracts.*

### Virtual Screening and Pharmacological Screening

**FIGURE 2:**
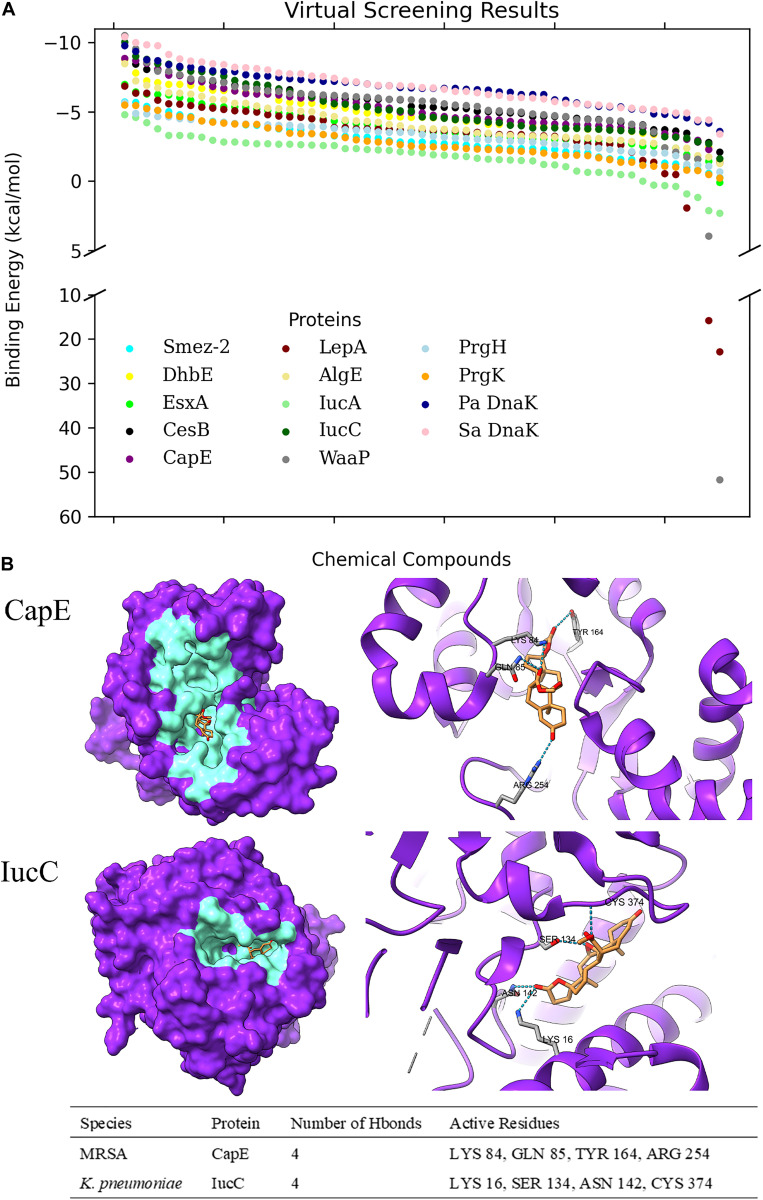
Virtual Screening of Chemical Determinants against selected target proteins. **(A)** Scatter plot of virtual screening through multiple bacterial virulent proteins based on their binding energies to each chemical determinant. Pa DnaK refers to *P. aeruginosa* DnaK protein, while Sa DnaK refers to *S. aureus* DnaK protein. **(B)** 3D representation of Eplerenone binding to CapE of MRSA and IucC of *K. pneumoniae*. The localization of active residues involved in binding and hydrogen bonding interactions are shown in the table format.

Among the total 65 chemical determinants obtained from different chromatographic analyses, 3 having chemical structure redundancies, and 7 which fall into the limitation of AutoDock 4, were discarded from further virtual screening pipeline to avoid inaccuracies in prediction parameterizations. Structure-based virtual screening of the rest 55 chemical determinants was conducted upon the top-ranked P2rank predicted druggable pockets (Figure 1 and Supplementary Table 2) of a set of 12 different bacterial key proteins from *S. enterica* (PrgK and PrgH), *K. pneumoniae* (IucA and IucC), *P. aeruginosa* (WaaP and AlgE), Methicillin-Resistant *S. aureus* (CapE and EsxA), *S. pyogenes* (LepA and SmeZ-2), and *B. subtilis* (DhbE and CesB) (Figure 2 and Supplementary Table 2). This has been utilized as the possible role players in determining the antibacterial activity of the *G. edulis* CEF. The complexes of these ligands with the proteins having binding energies around and lower than -7 kcal/mol indicate potentially druggable molecules or drug targets.

With a simultaneous consideration of the structure-based virtual screening and the predicted pharmacological properties of each chemical determinant, Eplerenone turned out to be a highly potentially druggable molecule. This is due to its strong binding affinity to most of the aforesaid proteins, which can be inferred from its moderate average binding score of -6.1007 kcal/mol. Moreover, it has a near-perfect predicted pharmacological properties for which adverse effects, due to ADME complications, would not be expected after intake.

Regarding Eplerenone, three target proteins, namely, CesB, CapE, and IucC, have manifested potential druggability due to stable binding energy lower than -7 kcal/mol *viz.* -7.66 kcal/mol, -7.21 kcal/mol, and -8.8 kcal/mol, respectively. The protein-ligand hydrogen bonding network has been observed for all three proteins (Figure 2). CesB, however, has been ceased for further analysis due to the absence of detectable protein-ligand hydrogen bonds. On the contrary, CapE and IucC have manifested rich intermolecular electrostatic interactions with Eplerenone having 4 hydrogen bonds each, and, therefore, was nominated to be potential drug targets of Eplerenone.

## Discussion

Resistance development of bacteria, to antibiotics, makes them ineffective, thereby resulting in the continuous demand for novel antibacterial agents. Marine organisms are potential sources of antibacterial agents that can probably inhibit the development of MDR bacteria. In the current study, we explored the antibacterial properties of Malaysian *G. edulis*, which are edible red seaweeds, largely found in Southeast Asian countries. Essentially, the *G. edulis* CEF, with various organic solvents, were screened against six MDR bacterial species, namely, *K. pneumoniae*, *P. aeruginosa, S. enterica*, methicillin-resistant *S. aureus* (MRSA), *S. pyogenes*, and *B. subtilis*. The screened CEF, showing promising antibacterial activities, were chromatographed through LC-MS and GC-MS to reveal a plethora of 65 compounds. A virtual screening of these compounds, followed by analyzing pharmacological properties, against selected key role playing proteins of the aforementioned bacteria, revealed Eplerenone as potential drug candidate for future pharmacological industries.

Among the set of six chosen solvents, in order of increasing polarity, i.e., chloroform (CF), ethyl acetate (EA), acetone (AC), ethanol (ET), methanol (MT), and water (WT), our findings confirmed that EA and AC sequential CEF were potentially effective in inhibiting the growth of all TBS. The remaining CEF responded either moderately or poorly, thereby providing a clear indication to proceed for further direct extraction from EA and AC. A moderate zone of inhibition was observed in EA and AC direct CEF against *B. subtilis* and *P. aeruginosa.* A concentration of 250 μg/mL was effective for EA and AC direct CEF against the TBS excluding *S. enterica* for this was 2000 μg/mL. On the contrary, however, in the case of sequential CEFs, EA displayed the inhibitory effect, at 2000 μg/ml, toward only *B. subtilis* and *S. enterica* whereas AC confirmed effect against *B. subtilis*, MRSA and *K. pneumoniae* at the same concentration.

Our findings revealed that EA and AC CEF are suitable for further exploration of the antibacterial properties of the red seaweed *G. edulis*. Our findings were more commensurate with related studies on the EA and AC CEF of red seaweeds, other than *G. edulis*. For instance, Salem et al. (2011) reported that the EA CEF of the red seaweed *Actinotrichia fragilis* showed positive antibacterial activity against *S. aureus, Bacillus cereus, E. coli, Salmonella* sp. and *P. aeruginosa.* Likewise, Abdel-Latif et al. (2018) confirmed that the, EA CEF of the red algae, *Grateloupia doryphora* had promising *in vitro* antibacterial potential toward *B. subtilis, E. faecalis, S. aureus, P. aeruginosa*, and *E. coli.* Again, Banu and Mishra (2018) reported the antibacterial effect of AC CEF of *Tricleocarpa fragilis* against *S. aureus* and *B. cereus.* However, there are few reports on the antibacterial potential of other solvents CEF of *G. edulis* as well. One of the earlier reports by Kolanjinathan et al. (2009) confirmed antibacterial potency of *G. edulis* ET CEF against *E. coli, Enterobacter aerogenes, S. aureus, P. aeruginosa*, and *Streptococcus faecalis*. Later, Kolanjinathan and Saranraj (2014) explored the antibacterial potential of MT CEF from *G. edulis* against, *S. pyogenes*, *B. subtilis, S. aureus, Staphylococcus epidermidis, B. cereus*, *K. pneumoniae*, *Salmonella enterica* serovar Typhi, *P. aeruginosa*, and *E. coli.* Recently, Umakanthan et al. (2017) described antibacterial effects of petroleum ether, hexane, AC, ET and MT CEF of *G. edulis* against *S. aureus* and *E. coli.* Thus, it turns out that our tested EA and AC CEF could reveal a better profile of the chemical compounds or metabolites present in *G. edulis*.

Based on the prominent antibacterial actions of the EA and AC CEF of *G. edulis*, we expected that both these CEF could harbour important bioactive compounds. Thus, sequential extracts of EA and AC CEF were subjected to a detailed mass-oriented LC-MS analysis (Supplementary Tables 6, 7). This revealed a widespread range of chemical classes existing in both the CEF. Amongst these, only 2 of the 22 compounds (with above 86% MFG scores) of the EA CEF have been reported to possess antibacterial activities (Supplementary Table 6). For example, the antibacterial effects of L13 (Gingerol) and L19 (Pheophorbide a) have been reported with by Prieto Rodríguez et al. (2011); Ghasemzadeh et al. (2016), respectively. Similarly, the AC extract fractions contained only 5, from a total of 29 compounds, with reported antibacterial activities (Supplementary Table 7). These are for L11 (6-Paradol) reported by El Dine et al. (2019), L22 (Carindone) by Lindsay et al. (2000), L23 (Pyropheophorbide a) by Kraatz et al. (2014), L24 (Pheophorbide-a) by Prieto Rodríguez et al. (2011), and L28 (Non-oxynol-9) by Hooton et al. (1991). Thus, possibilities exist for those 40 and 32 compounds from EA and AC fractions, respectively, with no matched identity with the library (Supplementary Tables 8, 9), to be therapeutically significant, though, further classification is obligatory to assess their usages.

Additional disclosure of important bioactive compounds was authenticated via GC-MS (Tables 3, 4). Notably, both sequential and direct CEF of EA and AC were subjected for GC-MS analysis. In contrast to the LC-MS reported compounds, about 95% of the chemical determinants, extracted through GC-MS, are unidentified for their antibacterial potentials. For example, among the 20 compounds from the sequential CEF of EA and AC (with area% scores above 0.5%) only 2 of the 12 compounds of the EA CEF have been reported with antibacterial properties (Table 3). These are SGEA2 reported by Padmavathi et al. (2014) and SGEA3 by Ghaneian et al. (2015). Along with 10 other compounds from EA CEF, the AC CEF contained 8 compounds without any *a priori* reported antibacterial activity (Table 3). Likewise, for the direct CEF of EA, only 1 out of 15 compounds detected (with area% scores above 0.5%) were known to possess such activity (Table 4). These are DGEA2 reported by Padmavathi et al. (2014), DGEA8 by Beavers et al. (2019), Again, for the AC CEF, 2 out of total 7 compounds detected, were reported with antibacterial effects (Table 4). These are DGAC6 by Marliyana et al. (2019) and DGAC7 by Padmavathi et al. (2014).

Over time, creditable progress in the field of virtual screening has permitted time- and cost-efficient drug discovery with repurposing (Abdella et al., 2020). Thus, we have considered a similar approach to antibacterial drug discovery from the CEF of Malaysian red seaweed, *G. edulis*. With a set of 65 compounds obtained through chromatographic analyses, we have conducted a series of computational analyses. This ensued with the selection of crucial target proteins from the TBS followed by a virtual screening of the set of chemicals onto the targets through molecular docking and finally analyzing their pharmacological properties to predict their bioavailability and toxicity (Supplementary Table 10). This culminated in the pinning of Eplerenone as the selected and predicted chemical compound from the EA and AC CEF of *G. edulis*. Importantly, Eplerenone is known as a potassium-sparing diuretic and has been used for patients with chronic heart diseases (CHD), but is more selective and thus, having fewer side effects, among the spironolactone class of steroidal antimineralocorticoids (Struthers et al., 2008; Pierson-Marchandise et al., 2017). As it is already in use in the market as a drug against CHD, it is imperative to have passed the clinical trials. Hence, it was not considered in this study to be further purified from the crude extracts and checked for cytotoxicity test (Tam et al., 2017). Thus, in essence, the proposal for the compound Eplerenone, for its antibacterial potential, reflects the concept of drug repurposing. It is important to note that, Eplerenone serves to be an alternative to antibiotics and is not proposed to be a new antibiotic *per se*.

To this end, our consideration of the proteins to be utilized for the aforementioned virtual screening also needs detailing in terms of their virulence potential. It is to be noted that virulence factors have gained traction over the years for considering the future plausible drug targets of MDR bacterial strains (Ogawara, 2021). Notably, without any imposition, on a bacterial population, for a highly selective pressure, anti-virulence drugs are better alternatives to antibiotics. Moreover, impaired virulence factors render the virulent pathogens to be pathobionts or avirulent bacteria. This might help the peaceful coexistence of the human population with these pathobionts. Thus, different representative virulent proteins from different bacteria were considered. These were a set of 12 different bacterial key proteins from *S. enterica* (PrgK and PrgH), *K. pneumoniae* (IucA and IucC), *P. aeruginosa* (WaaP and AlgE), methicillin-resistant *S. aureus* (CapE and EsxA), *S. pyogenes* (LepA and SmeZ-2) and *B. subtilis* (DhbE and CesB) (Figure 1). These proteins were selected due to their available crystal structure in PDB for further docking studies during virtual screening. On the contrary, earlier homology modelled and validated DnaK structure was utilized for *P. aeruginosa* and *S. aureus*. Essentially, DnaK acts as a molecular chaperone, mediated by its ATPase activities (Chiappori et al., 2015), and has been reported to be central in mediating bacterial stress responses due to its mutants exhibiting an increase in antimicrobial susceptibilities and decrease in survivability in the host (Wolska et al., 2000; Yamaguchi et al., 2003; Singh et al., 2007). Again, a study on the whole-genome analysis (WGA) of protein interaction network (PIN) reported that DnaK protein was crucial in mediating quorum sensing in multidrug resistant *Proteus mirabilis* (Pawar et al., 2018). Furthermore, WGA analyses of PIN from MDR pathogens like *P. aeruginosa*, *S. aureus*, *S. enterica*, *S. pneumoniae*, *P. mirabilis*, *Acinetobacter baumannii*, *Escherichia coli*, and *Mycobacterium tuberculosis* revealed DnaK to be among the top 10 crucial proteins indispensable for the cellular integrity of the bacteria (Mujawar et al., 2020). Hence, DnaK protein has also been selected for the *in silico* study, herein, as a promising drug target for selected MDR bacteria.

The proteins PrgK and PrgH, of *Salmonella* species, form the inner rings of the structurally complex syringe-like inject some, at the base substructure, which mediates the transfer of virulent factors into the host cell during invasion (Hu et al., 2017). IucA and IucC proteins are crucial mediators in the aerobactin synthesis pathway in bacteria (Mydy et al., 2020) and have been recently proposed as antivirulence targets in *K. pneumoniae* (Russo and Gulick, 2019). WaaP is a lipopolysaccharide heptose kinase, essential for *P. aeruginosa* growth due to its indispensable role in bacterial outer membrane synthesis by aiding in lipid-A core biosynthesis (Zhao and Lam, 2002). Again, in *Pseudomonas* species, the AlgE protein is essential in the alginate biosynthesis pathway which eventually leads to biofilm formation in bacteria and eventually results in virulence (Xu et al., 2019). To this end, we have also considered DnaK for *P. aeruginosa*. This protein is a bacterial molecular chaperone of the Heat Shock Protein 70 kDa (HSP70) family which mediates bacterial survival under stress conditions, especially antibiotic administration (Singh et al., 2012). It has been proposed as an attractive druggable target as well as a vaccine candidate (Fourie and Wilson, 2020). Moreover, DnaK has also been identified to be important in quorum sensing, as well as cellular integrity in bacteria by other studies (Pawar et al., 2018; Mujawar et al., 2020).

DnaK has also been considered for *S. aureus*, besides the CapE protein. The latter is involved in the biosynthesis of capsular polysaccharide (Miyafusa et al., 2013) which constitutes the cell surface carbohydrate layer to confer immunological and physiological protective effects to the bacteria thereby preventing phagocytic events and aiding in bacterial survival in host bloodstream. Moreover, the EsxA protein of *S. aureus* has an important role in establishing infections in the host by acting as adaptor proteins aiding protein transport during pathogenesis (Sundaramoorthy et al., 2008) and has been reported as a druggable target as well as a vaccine candidate in coping with *S. aureus* infections (Sze and Kao, 2020). LepA, being ranked 3rd in the aspect of the highest level of conservation in bacteria, acts as an elongation factor during the gene translation events in *S. pyogenes* (Evans et al., 2008) and has been reported to be crucial in aiding bacterial growth in a stressed environment, both chemically and physically (Heller et al., 2017). SmeZ, or Streptococcal mitogenic endotoxin Z, is the most potent superantigens as per the discovery thus far (Popugailo et al., 2019). It inhibits the inflammatory reactions in the host, as well as inducing immunological complications by acting on the crucial molecules of the immune system such as MHC-II and T cell receptor, thereby inducing T cell hyperactivation and eventually unnecessary tissue damage (Popugailo et al., 2019). The DhbE protein is involved in bacterial biosynthesis of siderophore thereby enabling the survival of bacteria like *Bacillus* species in high iron concentration environment, especially the blood (May et al., 2001). Again, in *Bacillus* species, the *cesB* gene encodes the protein cereulide (CesB), a notorious bacterial toxin with high heat and acid tolerance which has been haunting the food industry by eventuating foodborne diseases outbreaks (Ducrest et al., 2019; Rouzeau-Szynalski et al., 2020).

Cultivation of seaweed in a laboratory is not an easy task. Moreover, large-scale algae cultivation and production are affected by several prominent factors, such as light intensity, temperature, pH, salinity, nutrients availability, and the presence of oxygen, carbon dioxide and inorganic carbon. However, in Malaysia, large-scale cultivation of seaweeds is conducted at the open sea of the eastern coast of Sabah, which is situated below the monsoon and typhoon belt. Nevertheless, seaweeds possess a vast diversity of secondary metabolites, which are potential novel sources of bioactive constituents with a broad spectrum of biological actions. Moreover, some metabolites derived from seaweeds have not been found in terrestrial plants.

## Conclusion

The current study re-defines a method to reveal bioactive compounds from the crude extracts of Malaysian red seaweed *G. edulis*, having promising antibacterial activities against selected bacterial species. Three species of Gram-positive and -negative characters were remarkably inhibited by the sequential and direct extracts of ethyl acetate and acetone. These were further separated through chromatographic methods to reveal a plethora of chemical constituents to be considered for a downstream virtual screening against selected crucial proteins of the six bacteria. The additional pharmacological screening revealed Eplerenone with a potential to be repurposed as an alternate source for an antibacterial compound. While more studies are needed to establish such activity of Eplerenone, our metabolic profiling paves the way for future researchers to further explore such unseen potentials of the Malaysian natural product *G. edulis*.

## Data Availability Statement

The data presented in the study are deposited in the Metabolomics Workbench repository, http://dx.doi.org/10.21228/M8N11H.

## Author Contributions

CL conceived the concepts and planned and designed the analyses. AA extracted the natural product and assessed its antibacterial activity. AA analyzed the data for LC-MS and GC-MS with the help of CL. Y-YY provided the plant product and co-supervisory inputs. Y-CT and MS conducted the computational studies with occasional inputs from CL. AA and Y-CT generated the figures and tables with guidance provided and primarily wrote the manuscript aided by complete editorial upgradation by CL. All authors contributed to the article and approved the submitted version.

## Conflict of Interest

The authors declare that the research was conducted in the absence of any commercial or financial relationships that could be construed as a potential conflict of interest.

## References

[B1] Abdella M. Abdella B. Lahiri C. (2020). “Rediscovering and repurposing natural microbial macromolecules through computational approaches,” in *Microbial and Natural Macromolecules*, eds Das S. Dash H. R. (Amsterdam: Elsevier), 373–400.

[B2] Abdel-Latif H. H. Shams El-Din N. G. Ibrahim H. A. H. (2018). Antimicrobial activity of the newly recorded red alga *Grateloupia doryphora* collected from the Eastern Harbor, Alexandria, Egypt. *J. Appl. Microbiol.* 125 1321–1332. 10.1111/jam.14050 30047213

[B3] Agregán R. Munekata P. E. Domínguez R. Carballo J. Franco D. Lorenzo J. M. (2017). Proximate composition, phenolic content and in vitro antioxidant activity of aqueous extracts of the seaweeds *Ascophyllum nodosum*, *Bifurcaria bifurcata* and *Fucus vesiculosus*. effect of addition of the extracts on the oxidative stability of canola oil under accelerated storage conditions. *Int. Food. Res. J.* 99 986–994. 10.1016/j.foodres.2016.11.009 28865625

[B4] Arulkumar A. Rosemary T. Paramasivam S. Rajendran R. B. (2018). Phytochemical composition, in vitro antioxidant, antibacterial potential and GC-MS analysis of red seaweeds (*Gracilaria corticata* and *Gracilaria edulis*) from Palk Bay, India. *Biocatal. Agric. Biotechnol.* 15 63–71.

[B5] Asghar A. Tan Y. C. Zahoor M. Asnawi S. Yow Y.-Y. Khan E. (2020). *Chromatographic Analyses, Virtual Screening and Pharmacokinetics of Yellow Malaysian Rambutan (Nephelium lappaceum L.) Fruit Epicarp Extracts Reveal Potential Antibacterial Compounds.* Available online at 10.21203/rs.3.rs-125241/v1 (accessed January 14,2021).

[B6] Banu V. S. Mishra J. K. (2018). Antimicrobial activity of different solvent based crude extracts from red seaweed *Tricleocarpa fragilis* (L.) Huisman & RA Towns from the coast of South Andaman. *Pharma Innov.* 7 123–127.

[B7] Beavers W. N. Monteith A. J. Amarnath V. Mernaugh R. L. Roberts L. J. Chazin W. J. (2019). Arachidonic acid kills *Staphylococcus aureus* through a lipid peroxidation mechanism. *Mbio* 10:e01333–19. 31575763 10.1128/mBio.01333-19 PMC6775451

[B8] Berman H. M. Westbrook J. Feng Z. Gilliland G. Bhat T. N. Weissig H. (2000). The protein data bank. *Nucleic Acids Res.* 28 235–242. 10592235 10.1093/nar/28.1.235 PMC102472

[B9] Chan P. T. Matanjun P. Yasir S. M. Tan T. S. (2015). Antioxidant activities and polyphenolics of various solvent extracts of red seaweed, *Gracilaria changii*. *J. Appl. Phycol.* 27 2377–2386.

[B10] Cheesman M. J. Ilanko A. Blonk B. Cock I. E. (2017). Developing new antimicrobial therapies: are synergistic combinations of plant extracts/compounds with conventional antibiotics the solution? *Pharmacogn. Rev.* 11 57–72. 10.4103/phrev.phrev_21_17 28989242 PMC5628525

[B11] Cheung R. C. F. Wong J. H. Pan W. Chan Y. S. Yin C. Dan X. (2015). Marine lectins and their medicinal applications. *Appl. Microbiol. Biotechnol.* 99 3755–3773. 25794876 10.1007/s00253-015-6518-0 PMC7080081

[B12] Chiappori F. Fumian M. Milanesi L. Merelli I. (2015). DnaK as antibiotic target: hot spot residues analysis for differential inhibition of the bacterial protein in comparison with the human HSP70. *PLoS One* 10:e0124563. 10.1371/journal.pone.0124563 25905464 PMC4408060

[B13] Daina A. Michielin O. Zoete V. (2017). SwissADME: a free web tool to evaluate pharmacokinetics, drug-likeness and medicinal chemistry friendliness of small molecules. *Sci. Rep.* 7:42717. 10.1038/srep42717 28256516 PMC5335600

[B14] De Almeida C. L. Falcao Hde S. Lima G. R. Montenegro Cde A. Lira N. S. de Athayde-Filho P. F. (2011). Bioactivities from marine algae of the genus Gracilaria. *Int. J. Mol. Sci.* 12 4550–4573. 10.3390/ijms12074550 21845096 PMC3155369

[B15] Ducrest P. J. Pfammatter S. Stephan D. Vogel G. Thibault P. Schnyder B. (2019). Rapid detection of Bacillus ionophore cereulide in food products. *Sci. Rep.* 9:5814. 10.1038/s41598-019-42167-0 30967595 PMC6456620

[B16] Egan W. J. Merz K. M. Baldwin J. J. (2000). Prediction of drug absorption using multivariate statistics. *J. Med. Chem.* 43 3867–3877. 11052792 10.1021/jm000292e

[B17] El Dine R. S. Elfaky M. A. Asfour H. El Halawany A. M. (2019). Anti-adhesive activity of *Aframomum melegueta* major phenolics on lower respiratory tract pathogens. *Nat. Prod. Res.* 35 539–547. 10.1080/14786419.2019.1585843 31070056

[B18] El Shafay S. M. Ali S. S. El-Sheekh M. M. (2016). Antimicrobial activity of some seaweeds species from Red sea, against multidrug resistant bacteria. *Egypt. J. Aquat. Res.* 42 65–74.

[B19] Evans R. N. Blaha G. Bailey S. Steitz T. A. (2008). The structure of LepA, the ribosomal back translocase. *Proc. Natl. Acad. Sci. U.S.A.* 105 4673–4678. 10.1073/pnas.0801308105 18362332 PMC2290774

[B20] Fourie K. R. Wilson H. L. (2020). Understanding GroEL and DnaK stress response proteins as antigens for bacterial diseases. *Vaccines.* 8:773. 10.3390/vaccines8040773 PMC7767184 33348708

[B21] Ghaneian M. T. Ehrampoush M. H. Jebali A. Hekmatimoghaddam S. Mahmoudi M. (2015). Antimicrobial activity, toxicity and stability of phytol as a novel surface disinfectant. *Environ. Health. Eng. Manag.* 2 13–16.

[B22] Ghasemzadeh A. Jaafar H. Z. Rahmat A. (2016). Changes in antioxidant and antibacterial activities as well as phytochemical constituents associated with ginger storage and polyphenol oxidase activity. *BMC Compl. Alternative. Med.* 16:382. 10.1186/s12906-016-1352-1 27687000 PMC5043602

[B23] Ghose A. K. Viswanadhan V. N. Wendoloski J. J. (1999). A knowledge-based approach in designing combinatorial or medicinal chemistry libraries for drug discovery. 1. a qualitative and quantitative characterization of known drug databases. *J. Comb. Chem.* 1 55–68. 10.1021/cc9800071 10746014

[B24] Hann M. M. Keseru G. M. (2012). Finding the sweet spot: the role of nature and nurture in medicinal chemistry. *Nat. Rev. Drug Discov.* 11 355–365. 10.1038/nrd3701 22543468

[B25] Heller J. L. E. Kamalampeta R. Wieden H. J. (2017). Taking a step back from back-translocation: an integrative view of LepA/EF4’s cellular function. *Mol. Cell. Biol.* 37 e00653–16. 10.1128/MCB.00653-16 28320876 PMC5452718

[B26] Hemasudha T. S. Thiruchelvi R. Balashanmugam P. (2019). Antioxidant, antibacterial, and anticancer activity from marine red algae *Gracilaria Edulis*. *Asian J. Pharm. Clin. Res.* 12 276–279. 10.22159/ajpcr.2019.v12i2.29883

[B27] Hooton T. M. Fennell C. L. Clark A. M. Stamm W. E. (1991). Nonoxynol-9: differential antibacterial activity and enhancement of bacterial adherence to vaginal epithelial cells. *J. Infect. Dis.* 164 1216–1219. 10.1093/infdis/164.6.1216 1659602

[B28] Hu B. Lara-Tejero M. Kong Q. Galan J. E. Liu J. (2017). In situ molecular architecture of the *Salmonella* type. *Cell* 168 1065–1074.e10. 10.1016/j.cell.2017.02.022 28283062 PMC5393631

[B29] Ismail M. M. Gheda S. F. Pereira L. (2016). Variation in bioactive compounds in some seaweeds from Abo Qir bay, Alexandria, Egypt. *Rend. Lincei* 27 269–279.

[B30] Kasanah N. Amelia W. Mukminin A. Triyanto Isnansetyo A. (2019). Antibacterial activity of Indonesian red algae *Gracilaria edulis* against bacterial fish pathogens and characterization of active fractions. *Nat. Prod. Res.* 33 3303–3307. 10.1080/14786419.2018.1471079 29733690

[B31] Khelil-Radji F. Belhouari M. Chemlal-Kherraz D. Matallah-Boutiba A. Boutiba Z. (2017). Antimicrobial activity of aqueous and ethanol extracts of two marine algae collected from Algerian west coast. *Electron. J. Environ. Agric. Food Chem.* 6655 100–104.

[B32] Kolanjinathan K. Saranraj P. (2014). Pharmacological efficacy of marine seaweed *Gracilaria edulis* extracts against clinical pathogens. *Glob. J. Pharmacol.* 8 268–274.

[B33] Kolanjinathan K. Ganesh P. Govindarajan M. (2009). Antibacterial activity of ethanol extracts of seaweeds against fish bacterial pathogens. *Eur. Rev. Med. Pharmacol. Sci.* 13 173–177. 19673167

[B34] Kraatz M. Whitehead T. R. Cotta M. A. Berhow M. A. Rasmussen M. A. (2014). Effects of chlorophyll-derived efflux pump inhibitor pheophorbide a and pyropheophorbide a on growth and macrolide antibiotic resistance of indicator and anaerobic swine manure bacteria. *J. Antibiot.* 2014 1–14.

[B35] Krivák R. Hoksza D. (2018). P2Rank: machine learning based tool for rapid and accurate prediction of ligand binding sites from protein structure. *J Cheminformatics* 10:39. 10.1186/s13321-018-0285-8 PMC6091426 30109435

[B36] Lindsay E. A. Berry Y. Jamie J. F. Bremner J. B. (2000). Antibacterial compounds from Carissa lanceolata R.Br. *Phytochemistry* 55 403–406. 10.1016/s0031-9422(00)00343-5 11140600

[B37] Lipinski C. A. (2004). Lead- and drug-like compounds: the rule-of-five revolution. *Drug Discov. Today Technol.* 1 337–341. 10.1016/j.ddtec.2004.11.007 24981612

[B38] Liu B. Zheng D. Jin Q. Chen L. Yang J. (2019). VFDB 2019: a comparative pathogenomic platform with an interactive web interface. *Nucleic Acids Res.* 47 D687–D692. 10.1093/nar/gky1080 30395255 PMC6324032

[B39] Marliyana S. Wibowo F. Wartono M. Munasah G. (2019). Evaluation of antibacterial activity of sesquiterpene Ar-Turmerone from *Curcuma soloensis* Val. rhizome. *IOP Conf. Ser. Mater. Sci. Eng.* 578:012060.

[B40] May J. J. Wendrich T. M. Marahiel M. A. (2001). The dhb operon of bacillus subtilisEncodes the biosynthetic template for the catecholic siderophore 2, 3-dihydroxybenzoate-glycine-threonine trimeric ester bacillibactin. *J. Biol. Chem.* 276 7209–7217. 11112781 10.1074/jbc.M009140200

[B41] Mérillon J. M. Rivière C. (2018). *Natural Antimicrobial Agents.* Berlin: Springer.

[B42] Miyafusa T. Caaveiro J. M. Tanaka Y. Tsumoto K. (2013). Dynamic elements govern the catalytic activity of CapE, a capsular polysaccharide-synthesizing enzyme from *Staphylococcus aureus*. *FEBS Lett.* 587 3824–3830. 10.1016/j.febslet.2013.10.009 24157361

[B43] Morris G. M. Huey R. Lindstrom W. Sanner M. F. Belew R. K. Goodsell D. S. (2009). AutoDock4 and AutoDockTools4: automated docking with selective receptor flexibility. *J. Comput. Chem.* 30 2785–2791. 10.1002/jcc.21256 19399780 PMC2760638

[B44] Muegge I. Heald S. L. Brittelli D. (2001). Simple selection criteria for drug-like chemical matter. *J. Med. Chem.* 44 1841–1846. 10.1021/jm015507e 11384230

[B45] Mujawar S. Abd El-Aal A. A. A. Lahiri C. (2020). “Variant analysis from bacterial isolates affirms DnaK crucial for multidrug resistance,” in *International Work-Conference on Bioinformatics and Biomedical Engineering*, eds Rojas I. Valenzuela O. Rojas F. Herrera L. Ortuño F. (Berlin: Springer), 237–248.

[B46] Mydy L. S. Bailey D. C. Patel K. D. Rice M. R. Gulick A. M. (2020). The siderophore synthetase IucA of the aerobactin biosynthetic pathway uses an ordered mechanism. *Biochemistry* 59 2143–2153. 10.1021/acs.biochem.0c00250 32432457 PMC7325057

[B47] Ogawara H. (2021). Possible drugs for the treatment of bacterial infections in the future: anti-virulence drugs. *J. Antibiot.* 74 24–41. 10.1038/s41429-020-0344-z 32647212

[B48] Padmavathi A. R. Abinaya B. Pandian S. K. (2014). Phenol, 2,4-bis(1,1-dimethylethyl) of marine bacterial origin inhibits quorum sensing mediated biofilm formation in the uropathogen *Serratia marcescens*. *Biofouling* 30 1111–1122. 10.1080/08927014.2014.972386 25377484

[B49] Pawar S. Ashraf M. I. Mujawar S. Mishra R. Lahiri C. (2018). In silico identification of the indispensable quorum sensing proteins of multidrug resistant *Proteus mirabilis*. *Front. Cell Infect. Microbiol.* 8:269. 10.3389/fcimb.2018.00269 30131943 PMC6090301

[B50] Pettersen E. F. Goddard T. D. Huang C. C. Couch G. S. Greenblatt D. M. Meng E. C. (2004). UCSF Chimera—a visualization system for exploratory research and analysis. *J. Comput. Chem.* 25 1605–1612. 15264254 10.1002/jcc.20084

[B51] Pierson-Marchandise M. Gras V. Moragny J. Micallef J. Gaboriau L. Picard S. (2017). The drugs that mostly frequently induce acute kidney injury: a case− noncase study of a pharmacovigilance database. *Br. J. Clin. Pharmacol.* 83 1341–1349. 28002877 10.1111/bcp.13216 PMC5427222

[B52] Popugailo A. Rotfogel Z. Supper E. Hillman D. Kaempfer R. (2019). Staphylococcal and streptococcal superantigens trigger B7/CD28 costimulatory receptor engagement to hyperinduce inflammatory cytokines. *Front. Immunol.* 10:942. 10.3389/fimmu.2019.00942 31114583 PMC6503043

[B53] Prieto Rodríguez J. A. Patiño Ladino O. J. Lesmes L. Lozano J. M. Cuca Suárez L. E. (2011). Phytochemical study of *Uncaria guianensis* leaves and antibacterial activity evaluation. *Acta Amazon.* 41 303–310.

[B54] Rosemary T. Arulkumar A. Paramasivam S. Mondragon-Portocarrero A. Miranda J. M. (2019). Biochemical, micronutrient and physicochemical properties of the dried red seaweeds *Gracilaria edulis* and *Gracilaria corticata*. *Molecules* 24:2225. 10.3390/molecules24122225 31197120 PMC6630400

[B55] Rouzeau-Szynalski K. Stollewerk K. Messelhäusser U. Ehling-Schulz M. (2020). Why be serious about emetic *Bacillus cereus*: cereulide production and industrial challenges. *Food Microbiol.* 85:103279. 10.1016/j.fm.2019.103279 31500702

[B56] Russo T. A. Gulick A. M. (2019). Aerobactin synthesis proteins as antivirulence targets in hypervirulent *Klebsiella pneumoniae*. *ACS Infect. Dis.* 5 1052–1054. 31032610 10.1021/acsinfecdis.9b00117 PMC6625901

[B57] Salem W. M. Galal H. El-deen N. (2011). Screening for antibacterial activities in some marine algae from the red sea (Hurghada, Egypt). *Afr. J. Microbiol. Res.* 5 2160–2167.

[B58] Samdani A. Vetrivel U. (2018). POAP: a GNU parallel based multithreaded pipeline of open babel and AutoDock suite for boosted high throughput virtual screening. *Comput. Biol. Chem.* 74 39–48. 10.1016/j.compbiolchem.2018.02.012 29533817

[B59] Seedevi P. Moovendhan M. Viramani S. Shanmugam A. (2017). Bioactive potential and structural chracterization of sulfated polysaccharide from seaweed (*Gracilaria corticata*). *Carbohydr. Polym.* 155 516–524. 10.1016/j.carbpol.2016.09.011 27702543

[B60] Shrestha P. Cooper B. S. Coast J. Oppong R. Thuy N. D. T. Phodha T. (2018). Enumerating the economic cost of antimicrobial resistance per antibiotic consumed to inform the evaluation of interventions affecting their use. *Antimicrob. Resist. Infect. Control* 7:98. 10.1186/s13756-018-0384-3 PMC6085682 30116525

[B61] Singh V. K. Syring M. Singh A. Singhal K. Dalecki A. Johansson T. (2012). An insight into the significance of the DnaK heat shock system in *Staphylococcus aureus*. *Int. J. Med. Microbiol.* 302 242–252. 22748508 10.1016/j.ijmm.2012.05.001

[B62] Singh V. K. Utaida S. Jackson L. S. Jayaswal R. K. Wilkinson B. J. Chamberlain N. R. (2007). Role for dnaK locus in tolerance of multiple stresses in *Staphylococcus aureus*. *Microbiology* 153(Pt. 9), 3162–3173. 10.1099/mic.0.2007/009506-0 17768259

[B63] Struthers A. Krum H. Williams G. H. (2008). A comparison of the aldosterone-blocking agents eplerenone and spironolactone. *Clin. Cardiol.* 31 153–158. 10.1002/clc.20324 18404673 PMC6652937

[B64] Subermaniam K. Yow Y. Y. Lim S. H. Koh O. H. Wong K. H. (2020). Malaysian macroalga padina australis hauck attenuates high dose corticosterone-mediated oxidative damage in PC12 cells mimicking the effects of depression. *Saudi J. Biol. Sci.* 27 1435–1445. 10.1016/j.sjbs.2020.04.042 32489279 PMC7254034

[B65] Subramani R. Narayanasamy M. Feussner K. D. (2017). Plant-derived antimicrobials to fight against multi-drug-resistant human pathogens. *3 Biotech* 7:172. 10.1007/s13205-017-0848-9 28660459 PMC5489455

[B66] Sundaramoorthy R. Fyfe P. K. Hunter W. N. (2008). Structure of *Staphylococcus aureus* EsxA suggests a contribution to virulence by action as a transport chaperone and/or adaptor protein. *J. Mol. Biol.* 383 603–614. 18773907 10.1016/j.jmb.2008.08.047 PMC3465917

[B67] Sze K. H. Kao R. Y. T. (2020). Characterisation of *Staphylococcus aureus* virulence factor EsxA and structure-based screening of EsxA inhibitors for combating methicillin-resistant S aureus: abridged secondary publication. *Hong Kong Med. J.* 26(Suppl. 4), 35–38. 32690818

[B68] Tam T. S. Wu M. H. Masson S. C. Tsang M. P. Stabler S. N. Kinkade A. (2017). Eplerenone for hypertension. *Cochrane Database Syst. Rev.* 2:CD008996. 10.1002/14651858.CD008996.pub2 28245343 PMC6464701

[B69] Umakanthan G. Vinobaba P. Radampola K. (2017). Antioxidant activity of *Gracilaria edulis* (Rhodophyseae) in Sri Lanka. *Int. J. Res.* 4 1455–1472.

[B70] Veber D. F. Johnson S. R. Cheng H.-Y. Smith B. R. Ward K. W. Kopple K. D. (2002). Molecular properties that influence the oral bioavailability of drug candidates. *J. Med. Chem.* 45 2615–2623. 12036371 10.1021/jm020017n

[B71] Ventola C. L. (2015). The antibiotic resistance crisis: part 1: causes and threats. *P T.* 40 277–283. 25859123 PMC4378521

[B72] Wolska K. I. Bugajska E. Jurkiewicz D. Kuc M. Jozwik A. (2000). Antibiotic susceptibility of *Escherichia coli* dnaK and dnaJ mutants. *Microb. Drug Resist.* 6 119–126. 10.1089/107662900419429 10990266

[B73] World Health Organization [WHO] (2017). *WHO Publishes List of Bacteria for Which New Antibiotics are Urgently Needed.* Geneva: WHO. Available online at: http://www.who.int/mediacentre/news/releases/2017/bacteria-antibiotics-needed/en/ (accessed December 20, 2020).

[B74] Xu Z. Li K. Pan T. Liu J. Li B. Li C. (2019). Lonicerin, an anti-algE flavonoid against *Pseudomonas aeruginosa* virulence screened from Shuanghuanglian formula by molecule docking based strategy. *J. Ethnopharmacol.* 239:111909. 10.1016/j.jep.2019.111909 31026553

[B75] Yamaguchi Y. Tomoyasu T. Takaya A. Morioka M. Yamamoto T. (2003). Effects of disruption of heat shock genes on susceptibility of *Escherichia coli* to fluoroquinolones. *BMC Microbiol.* 3:16. 10.1186/1471-2180-3-16 12911840 PMC184496

[B76] Yap W. F. Tay V. Tan S. H. Yow Y. Y. Chew J. (2019). Decoding antioxidant and antibacterial potentials of malaysian green seaweeds: *Caulerpa racemosa* and *Caulerpa lentillifera*. *Antibiotics* 8:152. 10.3390/antibiotics8030152 31533237 PMC6783820

[B77] Zhao X. Lam J. S. (2002). WaaP of *Pseudomonas aeruginosa* is a novel eukaryotic type protein-tyrosine kinase as well as a sugar kinase essential for the biosynthesis of core lipopolysaccharide. *J. Biol. Chem.* 277 4722–4730. 11741974 10.1074/jbc.M107803200

