# Supplementary material for: Metabolite Profiling of Malaysian Gracilaria edulis Reveals Eplerenone as Novel Antibacterial Compound for Drug Repurposing Against MDR Bacteria
Source: Front Microbiol. 2021 Jun 30;12:653562. doi: 10.3389/fmicb.2021.653562 (PMC8279767; doi:10.3389/fmicb.2021.653562)
Supplement: Supplementary file 1 [file Data_Sheet_1.docx]

**Metabolite profiling of Malaysian *Gracilaria edulis* reveals Eplerenone as novel antibacterial compound for drug repurposing against MDR Bacteria**

**Authors:** Ali Asghar^1^, Muhammad Shahid^2^, Yong-Chiang Tan^1^, Yoon-Yen Yow^1^ and Chandrajit Lahiri^1*^

**Addresses:**

^1^ Department of Biological Sciences, Sunway University, Petaling Jaya, Malaysia

^2^ Department of Food Sciences, Universiti Kebangsaan, Bangi, Malaysia

**Correspondence:** *Chandrajit Lahiri, **E-mail:** [chandrajitl@sunway.edu.my](mailto:chandrajitl@sunway.edu.my)

**Phone:** +60 3-7491 8622**Fax:** +6 03 5638 7177

**Co-Authors:** Ali Asghar, E-mail: [ali.a13@imail.sunway.edu.my](mailto:ali.a13@imail.sunway.edu.my)

Muhammad Shahid, E-mail: [mshahdaslam@gmail.com](mailto:mshahdaslam@gmail.com)

Yong Chiang Tan, E-mail: [yong.t38@imail.sunway.edu.my](mailto:yong.t38@imail.sunway.edu.my)

Yoon-Yen Yow, E-mail: [yoonyeny@sunway.edu.my](mailto:yoonyeny@sunway.edu.my)

**SUPPLEMENTARY DATA**

**TABLE S1**. Antibiotic susceptibility profile of bacterial strains used in this study

| **N0** | **Drugs** | **Conc (µg)** | **Bacterial strains** | | | | | |
| --- | --- | --- | --- | --- | --- | --- | --- | --- |
|  |  |  | *B. subtilis* | MRSA | *S. pyogenes* | *P. aeruginosa* | *K. pneumonia* | *S. enterica* |
| 1 | Amoxicillin | 10 | R | R | R | R | R | R |
| 2 | Ampicillin | 10 | R | R | R | R | R | R |
| 3 | Ciprofloxacin | 10 | R | R | S | S | S | S |
| 4 | Colistin | 10 | R | R | I | S | R | S |
| 5 | Erythromycin | 15 | I | R | R | R | R | R |
| 6 | Gentamicin | 10 | S | S | S | S | S | S |
| 7 | Kanamycin | 30 | I | S | R | R | I | R |
| 8 | Norfloxacin | 10 | I | R | S | S | R | R |
| 9 | Tetracycline | 30 | I | S | R | R | R | S |
| 10 | Trimethoprim | 5 | R | S | I | R | S | S |

S: Susceptible, R: Resistant, I: intermediate

**TABLE S2**. P2Rank Predicted Druggable Pockets.

|  |  | **PDB Structure** | | **P2Rank**  **Pocket Residues** |
| --- | --- | --- | --- | --- |
| **Species** | **Protein Name** | **PDB ID** | **Chain ID** |  |
| ***Salmonella enterica*** | PrgK | 6UOT | Y | 102, 106, 109, 133, 143, 144, 146, 175, 176 |
|  | PrgH | 6UOT | A | 226, 229, 230, 233, 245, 246, 247, 347, 352, 353 |
| ***Klebsiella pneumoniae*** | IucA | 5JM8 | A | 10, 11, 12, 15, 16, 19, 23, 54, 60, 61, 62, 143, 145, 146, 147, 148, 149, 153, 154, 168, 261, 262, 263, 264, 265, 276, 283, 284, 285, 286, 288, 297, 363, 366, 367, 368, 369, 372, 373, 375, 423, 425, 426, 427, 428, 444, 445, 447, 448, 450, 471, 475, 479, 482, 483, 486, 487, 491, 495, 498, 499, 540, 541, 542, 563 |
|  | IucC | 6CN7 | A | 8, 9, 12, 13, 15, 16, 20, 57, 59, 60, 61,106, 109, 110, 113, 134, 135, 137, 142, 372, 373, 374, 375, 376, 378, 491, 494, 495, 498 |
| ***Pseudomonas aeruginosa*** | WaaP | 6DFL | A | 123, 127, 130, 143, 144, 147, 166, 207, 210, 211, 214, 217, 219, 224, 227, 228, 247, 250, 251 |
|  | AlgE | 5D5D | A | 45, 74, 76, 78, 80, 83, 85, 87, 89,129, 130, 132, 134, 136, 142, 145, 147, 151, 152, 154, 155, 156, 157, 159, 160, 161, 169, 171, 173, 178, 180, 210, 212, 214, 218, 220, 222, 224, 254, 258, 260, 262, 263, 264, 265, 275, 276, 277, 279, 312, 313, 344, 346, 347, 374, 489, 490 |
|  | DnaK | *homology model | | 8, 10, 11, 12, 13, 37, 39, 55, 58, 65, 67, 68, 70, 171, 194, 196, 197, 199, 229, 230, 233, 260, 263, 264, 267, 270, 271, 274, 341, 342, 343, 345, 367, 368, 371 |
| ***Bacillus subtilis*** | DhbE | 1MDB | A | 189, 190, 191, 192, 193, 194, 195, 196, 197, 198, 234, 235, 236, 240, 280, 307, 308, 309, 311, 329, 330, 331, 332, 333, 334, 392, 398, 401, 402, 409, 410, 411, 412, 413, 425, 426, 427, 428, 430, 431, 442, 443, 446, 450, 457, 458, 519, 520, 522 |
|  | CesB | 4CCY | A | 63, 64, 65, 66, 90, 91, 98,100, 101, 102, 103, 108, 130, 131, 135, 156, 162, 166, 167, 170, 173, 182, 210, 211, 213, 214, 215, 216, 217, 221, 222, 247, 274 |
| ***Staphylococcus aureus*** | CapE | 4G5H | A | 11, 13, 14, 15, 16, 17, 35, 36, 37, 38, 41, 59, 60, 61, 80, 81, 82, 83, 84, 85, 86, 87, 99, 122, 123, 124, 126, 134, 138, 164, 165, 166, 167, 168, 170, 172, 173, 174, 175, 177, 189, 191, 195, 197, 198, 199, 202, 229, 231, 234, 251, 254, 257 |
|  | EsxA | 2VS0 | A | 29, 32, 55, 58, 59, 62 |
|  | DnaK | *homology model | | 8, 10, 11, 12, 13, 14, 15, 35, 145, 168, 170, 171, 173, 199, 200, 237, 240, 241, 311, 312, 313, 315, 337, 338 |
| ***Streptococcus pyogenes*** | LepA | 4K8W | A | 70, 71, 86, 87, 88, 103, 105, 110, 112, 113, 116, 139 |
|  | SmeZ-2 | 1EU3 | A | 26, 27, 29, 32, 34, 49, 82, 84, 86 |

**TABLE S3**. Search Grid Parameters for Virtual Screening.

|  | **Centre** | | | **Grid Points** | | |
| --- | --- | --- | --- | --- | --- | --- |
| **Proteins** | **x** | **y** | **z** | **x** | **y** | **z** |
| 6UOT_Y | 211.15 | 228.064 | 223.557 | 17 | 21 | 19 |
| 6UOT_A | 256.102 | 242.187 | 220.716 | 17 | 14 | 17 |
| 5JM8_A | 59.8722 | 76.9042 | 65.3801 | 25 | 37 | 20 |
| 6CN7_A | -19.3974 | 186.232 | 74.6408 | 28 | 21 | 24 |
| 6DFL_A | -46.5216 | 30.3051 | 31.0402 | 21 | 17 | 20 |
| 5D5D_A | 137.836 | 148.924 | 10.9762 | 31 | 32 | 34 |
| PaDNAK | 18.069 | 74.299 | 28.532 | 24 | 24 | 24 |
| 1MDB_A | 0.7384 | 24.6479 | 38.2415 | 28 | 29 | 30 |
| 4CCY_A | 49.5971 | 1.37983 | 42.4946 | 33 | 23 | 23 |
| 4G5H_A | -43.2312 | 12.3613 | 27.8621 | 27 | 31 | 33 |
| 2VS0_A | 28.7939 | 25.653 | 13.3844 | 21 | 13 | 17 |
| SaDNAK | 17.647 | 75.43 | 27.766 | 24 | 24 | 24 |
| 4K8W_A | 11.9209 | 14.3784 | 16.5007 | 20 | 16 | 18 |
| 1EU3_A | 2.88424 | 26.3501 | -15.8185 | 23 | 16 | 24 |

***Note*:** PaDNAK refers to *P. aeruginosa* dnaK protein, SaDNAK refers to *S. aureus* dnaK protein.

**TABLE S4.** Antibacterial potential of *G. edulis* sequential extracts based on disc diffusion assay

| **Microorganisms** | **Zones of Inhibition (mm)** | | | | | |
| --- | --- | --- | --- | --- | --- | --- |
|  | **CF** | **ET** | **MT** | **WT** | **SC** | **PC** |
| ***B. subtilis*** | - | - | - | - | - | 32.00 **±**  0.70 |
| **MRSA** | - | - | - | - | - |  |
| ***S. pyogenes*** | - | - | - | - | - |  |
| ***P. aeruginosa*** | - | - | - | - | - |  |
| ***K. pneumonia*** | - | - | - | - | - |  |
| ***S. enterica*** | - | - | - | - | - |  |

“-” no activity, P.C: positive control (Gentamicin 10 µg), SC: solvent control (DMSO <1%), CF: chloroform, ET: ethanol, MT: methanol; WT: water extract. The data is expressed as the mean ±standard error of two independent experiments performed in technical triplicates.

**TABLE S5**. MIC of the G. edulis sequential and direct extracts against all tested pathogens.

| **Extracts** | **Minimum Inhibitory Concentration (MIC) (μg/mL** | | | | | |
| --- | --- | --- | --- | --- | --- | --- |
|  | ***MRSA*** | ***B. subtilis*** | ***S. pyogenes*** | ***P. aeruginosa*** | ***S. enterica*** | ***K. pneumonia*** |
| **CF** | ND | ND | ND | ND | ND | ND |
| **ET** | ND | ND | ND | ND | ND | ND |
| **MT** | ND | ND | ND | ND | ND | ND |
| **WT** | ND | ND | ND | ND | ND | ND |

ND: not determined, CF; Chloroform, EA; ethyl acetate, AC; acetone, ET; ethanol, MT; methanol, WT; water. The illustrated MIC values are the lowest inhibitory concentrations achieved from two independent trials performed in triplicate

**Exploration of other chemical determinants through Liquid Chromatography–Mass Spectrometry (LC-MS) Analysis**

**TABLE S6.** Identified compounds existing in *G. edulis* ethyl acetate extract by LC-MS analysis

| **No** | **m/z** | **Identified compounds** | **Molecular formula** | **RT (min)** | **Mass** | **Score (DB)** | **Antibacterial activity**  **report** |
| --- | --- | --- | --- | --- | --- | --- | --- |
| 1 | 113.1324 | cis-1,2-dimethylcyclohexane | C_8_ H_16_ | 24.666 | 112.125 | 86.54 | NR |
| 2 | 137.0807 | 3-Deoxy-D-arabinitol | C_5_ H_12_ O_4_ | 12.75 | 136.0734 | 87.04 | NR |
| 3 | 139.1113 | 6,8-nonadienal | C_9_ H_14_ O | 12.579 | 138.104 | 96.77 | NR |
| 4 | 141.091 | 4-Vinylcyclohexene diepoxide | C_8_ H_12_ O_2_ | 11.743 | 140.0837 | 86.04 | NR |
| 5 | 157.1221 | 2E-Heptenyl acetate | C_9_ H_16_ O_2_ | 12.582 | 156.1149 | 98.08 | NR |
| 6 | 181.1222 | 3-tert-Butyl-5-methylcatechol | C_11_ H_16_ O_2_ | 15.188 | 180.1148 | 99.5 | NR |
| 7 | 197.1143 | (+)-3-hydroxy pelargonic acid | C_9_ H_18_ O_3_ | 9.729 | 174.1251 | 86.9 | NR |
| 8 | 197.1169 | 4-(2-hydroxypropoxy)-3,5-dimethyl-Phenol | C_11_ H_16_ O_3_ | 11.754 | 196.1097 | 99.39 | NR |
| 9 | 274.2736 | C16 Sphinganine | C_16_ H_35_ N O_2_ | 14.201 | 273.266 | 88.15 | NR |
| 10 | 275.2013 | 3Z,6Z,9Z,12Z,15Z-octadecapentaenoic acid | C_18_ H_26_ O_2_ | 19.715 | 274.1942 | 89.8 | NR |
| 11 | 279.1596 | Emmotin A | C_16_ H_22_ O_4_ | 19.799 | 278.1523 | 98.73 | NR |
| 12 | 285.2226 | 13-cis-retinal | C_20_ H_28_ O | 21.468 | 284.2152 | 89.85 | NR |
| 13 | 295.1913 | Gingerol | C_17_ H_26_ O_4_ | 15.357 | 294.1839 | 93.58 | Reported by Ghasemzadeh *et al.* 2016. |
| 14 | 303.2317 | 5(S)-HETE lactone | C_20_ H_30_ O_2_ | 18.768 | 302.2245 | 99.25 | NR |
| 15 | 317.2116 | Cyrneine A | C_20_ H_28_ O_3_ | 15.543 | 316.2046 | 92.47 | NR |
| 16 | 331.2643 | 10-[5]-ladderane-decanoic acid | C_22_ H_34_ O_2_ | 21.583 | 330.2573 | 92.06 | NR |
| 17 | 335.2218 | 7beta-Hydroxy-lathyrol | C_20_ H_30_ O_4_ | 16.933 | 334.2148 | 95.82 | NR |
| 18 | 371.2583 | 3-Oxo-5β-chola-7,9(11)-dien-24-oic Acid | C_24_ H_34_ O_3_ | 21.581 | 370.2508 | 95.37 | NR |
| 19 | 593.2756 | Pheophorbide a | C_35_ H_36_ N_4_ O_5_ | 23.539 | 592.2686 | 98.44 | Reported by Prieto Rodríguez *et al.,* 2011. |
| 20 | 594.3997 | Discodermolide | C_33_ H_55_ N O_8_ | 20.084 | 593.3924 | 99.62 | NR |
| 21 | 599.3558 | 1-Hydroxyvitamin D3 3-D-glucopyranoside | C_33_ H_52_ O_8_ | 20.083 | 576.3666 | 97.89 | NR |
| 22 | 609.2709 | Harderoporphyrin | C_35_ H_36_ N_4_ O_6_ | 22.305 | 608.2636 | 99.15 | NR |

**TABLE S7.** Identified compounds existing in *G. edulis* acetone extract by LC-MS analysis

| **No** | **m/z** | **Identified compounds** | **Molecular formula** | **RT (min)** | **Mass** | **Score (DB)** | **Antibacterial activity**  **report** |
| --- | --- | --- | --- | --- | --- | --- | --- |
| 1 | 113.133 | cis-1,2-dimethylcyclohexane | C_8_ H_16_ | 24.68 | 112.13 | 87.87 | NR |
| 2 | 181.122 | 3-tert-Butyl-5-methylcatechol | C_11_ H_16_ O_2_ | 15.19 | 180.11 | 99.8 | NR |
| 3 | 197.117 | 4-(2-hydroxypropoxy)-3,5-dimethyl-Phenol | C_11_ H_16_ O_3_ | 11.76 | 196.11 | 98.86 | NR |
| 4 | 203.091 | Diethyl Oxalpropionate | C_9_ H_14_ O_5_ | 8.913 | 202.08 | 95.14 | NR |
| 5 | 274.274 | C16 Sphinganine | C_16_ H_35_ N O_2_ | 14.2 | 273.27 | 96.59 | NR |
| 6 | 275.201 | 3Z,6Z,9Z,12Z,15Z-octadecapentaenoic acid | C_18_ H_26_ O_2_ | 19.72 | 274.19 | 89.94 | NR |
| 7 | 277.179 | Onchidal | C_17_ H_24_ O_3_ | 15.36 | 276.17 | 86.14 | NR |
| 8 | 279.16 | Emmotin A | C_16_ H_22_ O_4_ | 19.81 | 278.15 | 97.73 | NR |
| 9 | 285.222 | 13-cis-retinal | C_20_ H_28_ O | 21.47 | 284.22 | 94.09 | NR |
| 10 | 290.269 | 2-Hydroxyhexadecanoic acid | C_16_ H_32_ O_3_ | 14.37 | 272.24 | 98.24 | NR |
| 11 | 296.222 | 6-Paradol | C_17_ H_26_ O_3_ | 17.58 | 278.19 | 88.47 | Reported (El Dine *et al.* 2019) |
| 12 | 303.232 | 5(S)-HETE lactone | C_20_ H_30_ O_2_ | 18.77 | 302.22 | 99.31 | NR |
| 13 | 305.247 | (-)-Isoamijiol | C_20_ H_32_ O_2_ | 19.37 | 304.24 | 96.03 | NR |
| 14 | 306.103 | Pirimiphos-methyl | C_11_ H_20_ N_3_ O_3_ P S | 18.69 | 305.1 | 96.66 | NR |
| 15 | 317.211 | Cyrneine A | C_20_ H_28_ O_3_ | 15.54 | 316.2 | 91.78 | NR |
| 16 | 331.264 | 10- [5]-ladderane-decanoic acid | C_22_ H_34_ O_2_ | 21.32 | 330.26 | 93.87 | NR |
| 17 | 371.258 | 3-Oxo-5β-chola-7,9(11)-dien-24-oic Acid | C_24_ H_34_ O_3_ | 21.47 | 370.25 | 96.9 | NR |
| 18 | 374.341 | N-propyl α, α-dimethylarachidonoyl amine | C_25_ H_43_ N O | 23.48 | 373.33 | 94.66 | NR |
| 19 | 415.212 | Eplerenone | C_24_ H_30_ O_6_ | 16.63 | 414.2 | 92.18 | NR |
| 20 | 460.343 | (22S)-1α,22,25-trihydroxy-23,24-tetradehydro-24a-homo-20-epivitamin D3 / (22S)-1α,22,25-trihydroxy-23,24-tetradehydro-24a-homo-20-epicholecalciferol | C_28_ H_42_ O_4_ | 23.52 | 442.31 | 89.91 | NR |
| 21 | 502.374 | (25S)-5alpha-cholestan-3beta,6alpha,7beta,8beta,15alpha,16beta,26-heptol | C_27_ H_48_ O_7_ | 20.41 | 484.34 | 98.25 | NR |
| 22 | 530.349 | Carindone | C_31_ H_44_ O_6_ | 20.87 | 512.32 | 94.13 | Reported (Lindsay *et al.* 2000) |
| 23 | 535.271 | Pyropheophorbide a | C_33_ H_34_ N_4_ O_3_ | 24.53 | 534.26 | 96.82 | Reported (Kraatz *et al.* 2014) |
| 24 | 593.277 | Pheophorbide a | C_35_ H_36_ N_4_ O_5_ | 22.89 | 592.27 | 97.85 | Reported (Prieto Rodríguez *et al.,* 2011) |
| 25 | 594.401 | Discodermolide | C_33_ H_55_ N O_8_ | 20.09 | 593.39 | 97.6 | NR |
| 26 | 599.356 | 1-Hydroxyvitamin D3 3-D-glucopyranoside | C_33_ H_52_ O_8_ | 20.09 | 576.37 | 91.83 | NR |
| 27 | 609.271 | Harderoporphyrin | C_35_ H_36_ N_4_ O_6_ | 22.32 | 608.26 | 99.67 | NR |
| 28 | 634.452 | Nonoxynol-9 | C_33_ H_60_ O_10_ | 20.2 | 616.42 | 97.74 | Reported (Hooton *et al.* 1991) |
| 29 | 653.297 | Haplophytine | C_37_ H_40_ N_4_ O_7_ | 24.32 | 652.29 | 98.49 | NR |

**LC-MS data of Metlin-Unmatched Compounds from *N. lappaceum* Sequential Extract.**

**TABLE S8.** Molecular Formula of Metlin-Unmatched Compounds from *G. edulis* ethyl acetate extract.

| **NO** | **Molecular Formula** | **RT (Min)** | **m/z** | **Mass** |
| --- | --- | --- | --- | --- |
|  | C_8_ H_4_ O_3_ | 24.679 | 149.0237 | 148.0164 |
|  | C_9_ H_19_ N O | 15.307 | 158.154 | 157.1467 |
|  | C_6_ H_2_ N_2_ O_3_ S | 1.362 | 182.9853 | 181.979 |
|  | C_11_ H_18_ O_3_ | 15.132 | 199.1335 | 198.1265 |
|  | C_10_ H_12_ N_4_ O_2_ | 8.912 | 221.102 | 220.0956 |
|  | C_13_ H_25_ N O_2_ | 14.94 | 228.1962 | 227.1887 |
|  | C_16_ H_34_ O_3_ | 21.544 | 275.2588 | 274.2513 |
|  | C_20_ H_22_ N | 17.687 | 277.1823 | 276.1751 |
|  | C_17_ H_36_ O_3_ | 22.177 | 289.2741 | 288.2664 |
|  | C_14_ H_30_ O_4_ S | 17.686 | 295.1922 | 294.1859 |
|  | C_16_ H_36_ N_3_ O_2_ | 23.104 | 303.2892 | 302.2807 |
|  | C_22_ H_28_ N O | 20.828 | 323.2249 | 322.218 |
|  | C_23_ H_34_ N | 22.437 | 325.2761 | 324.2686 |
|  | C_20_ H_38_ N S | 23.77 | 325.2799 | 324.2727 |
|  | C_19_ H_40_ O_2_ S | 21.984 | 333.281 | 332.2743 |
|  | C_18_ H_38_ N O_4_ | 22.093 | 333.287 | 332.2799 |
|  | C_19_ H_34_ N_4_ | 21.513 | 336.3131 | 318.2791 |
|  | C_22_ H_30_ O_3_ | 18.763 | 343.2245 | 342.2189 |
|  | C_18_ H_26_ N_6_ O | 18.952 | 343.2247 | 342.2164 |
|  | C_17_ H_41_ N_4_ O_3_ | 22.198 | 350.3244 | 349.317 |
|  | C_21_ H_40_ O_2_ S | 20.973 | 357.2826 | 356.275 |
|  | C_21_ H_38_ N_3_ S | 23.696 | 365.2855 | 364.2783 |
|  | C_19_ H_36_ N_7_ | 21.5 | 380.3371 | 362.3031 |
|  | C_22_ H_22_ N_6_ O | 16.349 | 387.1934 | 386.1863 |
|  | C_19_ H_41_ N_11_ | 21.491 | 424.3632 | 423.3552 |
|  | C_26_ H_26_ N_3_ O_3_ | 18.009 | 429.2053 | 428.1981 |
|  | C_24_ H_41_ N_8_ O | 20.439 | 458.3478 | 457.3404 |
|  | C_23_ H_47_ N_8_ O_2_ | 21.464 | 468.3901 | 467.3822 |
|  | C_30_ H_57_ N O_3_ S | 21.428 | 512.4153 | 511.4075 |
|  | C_26_ H_35_ N_2_ O_2_ S_4_ | 23.195 | 536.165 | 535.1573 |
|  | C_27_ H_53_ N_4_ O_7_ | 20.342 | 546.3991 | 545.3919 |
|  | C_32_ H_28_ N_2_ S_3_ | 22.252 | 554.1753 | 536.141 |
|  | C_25_ H_53_ N_11_ O_3_ | 21.382 | 556.4413 | 555.4336 |
|  | C_34_ H_57_ N_2_ O_6_ | 20.273 | 590.4256 | 589.4216 |
|  | C_37_ H_38_ N O_8_ | 22.36 | 625.2668 | 624.2595 |
|  | C_35_ H_37_ N_3_ S_4_ | 23.442 | 628.1947 | 627.1869 |
|  | C_37_ H_40_ N_4_ O_6_ | 24.411 | 637.302 | 636.2949 |
|  | C_28_ H_42_ N_7_ O_10_ | 21.539 | 654.3324 | 636.2993 |
|  | C_35_ H_67_ N O_11_ | 20.128 | 678.4782 | 677.4708 |
|  | C_37_ H_71_ N O_12_ | 20.056 | 722.5051 | 721.4974 |

**TABLE S9.** Molecular Formula of Metlin-Unmatched Compounds from *G. edulis* acetone extract.

| **NO** | **Formula** | **RT (Min)** | **m/z** | **Mass** |
| --- | --- | --- | --- | --- |
|  | C_7_ H_12_ N_3_ | 9.729 | 139.1109 | 138.1037 |
|  | C_8_ H_4_ O_3_ | 19.8 | 149.023 | 148.0157 |
|  | C_7_ H_14_ N_3_ O | 9.731 | 157.1212 | 156.114 |
|  | C_9_ H_19_ N O | 15.308 | 158.1539 | 157.1466 |
|  | C_6_ H_2_ N_2_ O_3_ S | 1.356 | 182.9851 | 181.9792 |
|  | C_18_ H_26_ N_8_ S | 6.509 | 194.1073 | 386.2006 |
|  | C_11_ H_18_ O_3_ | 15.134 | 199.1335 | 198.1262 |
|  | C_13_ H_25_ N O_2_ | 14.933 | 228.1957 | 227.1883 |
|  | C_16_ H_34_ O_3_ | 21.536 | 275.2589 | 274.2514 |
|  | C_20_ H_22_ N | 17.684 | 277.1831 | 276.1757 |
|  | C_17_ H_36_ O_3_ | 22.541 | 289.2743 | 288.2663 |
|  | C_18_ H_38_ O_3_ | 23.089 | 303.29 | 302.2818 |
|  | C_23_ H_34_ N | 22.421 | 325.2762 | 324.2686 |
|  | C_20_ H_38_ N S | 23.72 | 325.279 | 324.272 |
|  | C_25_ H_32_ N | 22.153 | 347.2604 | 346.2532 |
|  | C_20_ H_39_ N5 | 22.173 | 350.3268 | 349.3196 |
|  | C_21_ H_38_ N_3_ S | 23.653 | 365.2848 | 364.2778 |
|  | C_15_ H_22_ N_4_ O S_3_ | 22.242 | 371.1027 | 370.0952 |
|  | C_24_ H_24_ N_3_ O_2_ | 16.352 | 387.194 | 386.187 |
|  | C_22_ H_40_ N_4_ S | 21.9 | 393.3025 | 392.2969 |
|  | C_21_ H_43_ N_8_ O | 21.482 | 424.363 | 423.3555 |
|  | C_29_ H_22_ N_2_ S_2_ | 21.039 | 463.1306 | 462.1226 |
|  | C_36_ H_21_ Cl N O | 22.242 | 519.1398 | 518.1322 |
|  | C_29_ H_32_ N_2_ S_4_ | 22.242 | 554.1781 | 536.1436 |
|  | C_28_ H_17_ N_11_ S | 22.243 | 557.1769 | 539.1386 |
|  | C_38_ H_40_ N O_7_ | 23.769 | 623.2878 | 622.2809 |
|  | C_35_ H_36_ N_4_ O_7_ | 22.348 | 625.2657 | 624.258 |
|  | C_37_ H_38_ N O_8_ | 20.654 | 625.267 | 624.2594 |
|  | C_35_ H_34_ N_2_ S_4_ | 23.43 | 628.1957 | 610.1611 |
|  | C_33_ H_20_ N_13_ O_2_ | 23.43 | 631.1944 | 630.1856 |
|  | C_39_ H_42_ N O_7_ | 24.397 | 637.3032 | 636.2957 |
|  | C_32_ H_52_ N_15_ O | 24.234 | 663.4549 | 662.4479 |


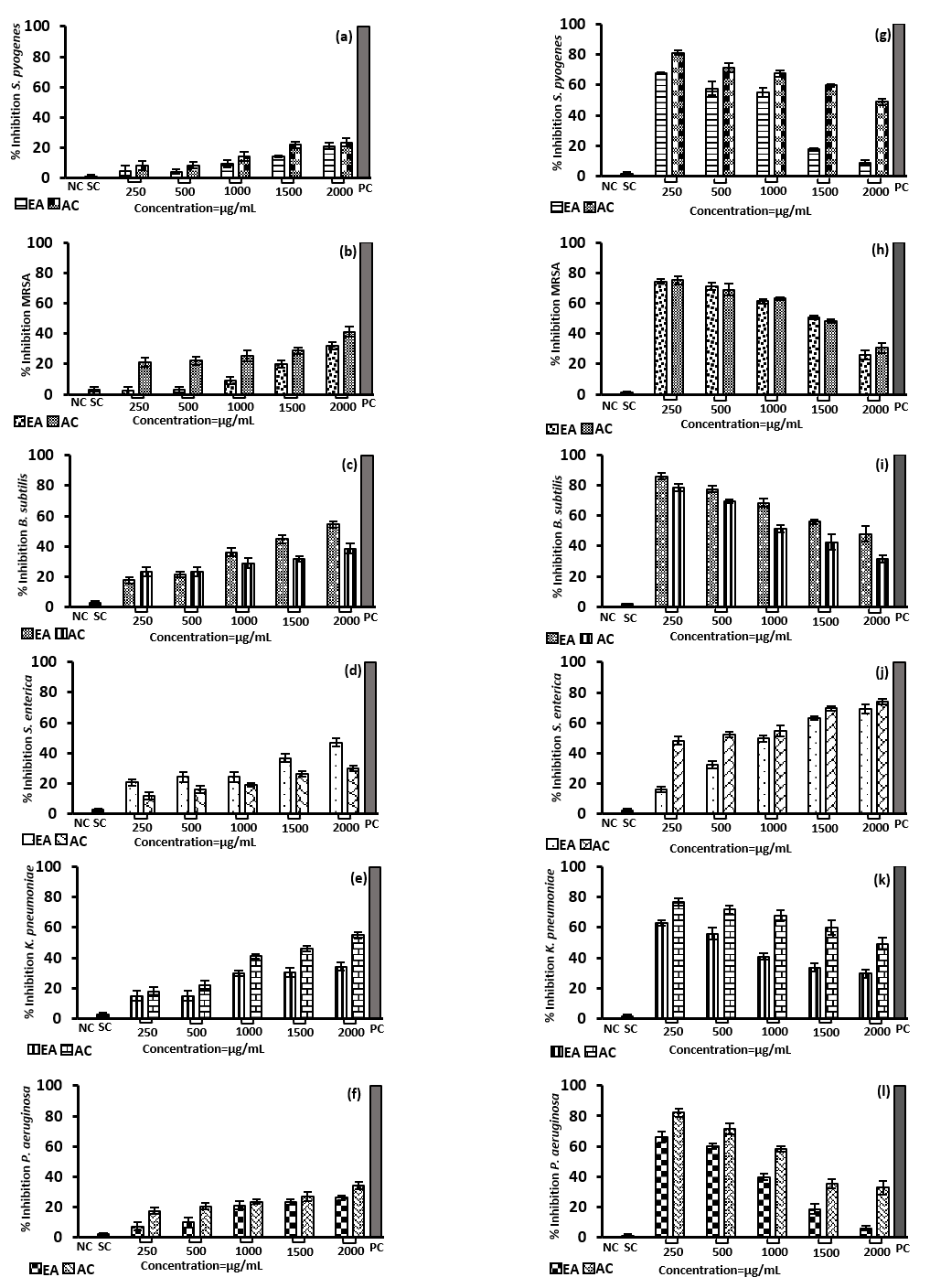
**Potential *in-vitro* antibacterial activites of Malaysian red seaweed (*G. edulis*) extracts.**

**FIGURE S1.** Percentage antibacterial activity of *G. edulis* sequential and direct extracts via broth dilution method. EA: ethyl acetate extract, AC: acetone extract, SC: solvent control (DMSO), NC: negative control, (only bacterial) PS: Positive control (Gentamicin, 10µg/mL).The crude (sequential; **a-f**, and direct; **g-l**) fractions were screened against gram-positive and gram-negative pathogens. **(a, g)** *S. pyogenes* **(b, h)** MRSA. **(c, i)** *B. subtilis.* **(d, j)** *S. enterica.* **(e, k)** *K. pneumoniae.* **(f, l)** *P. aeruginosa.* The results are demonstrative of some experiments achieved in triplicate and expressed as the mean ± standard error.


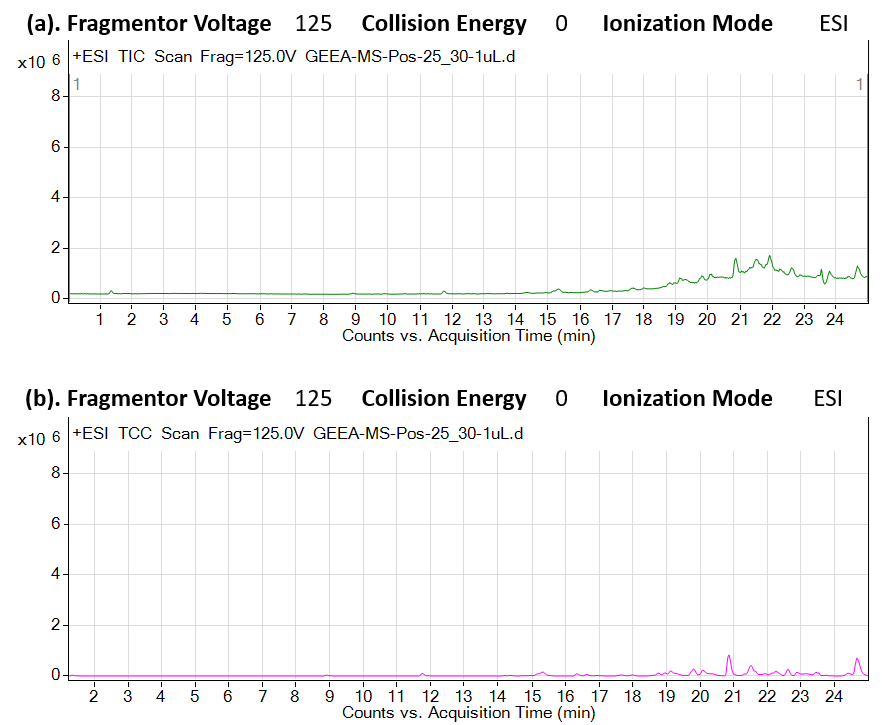
**LC-MS analysis of *G. edulis* sequential extracts:**


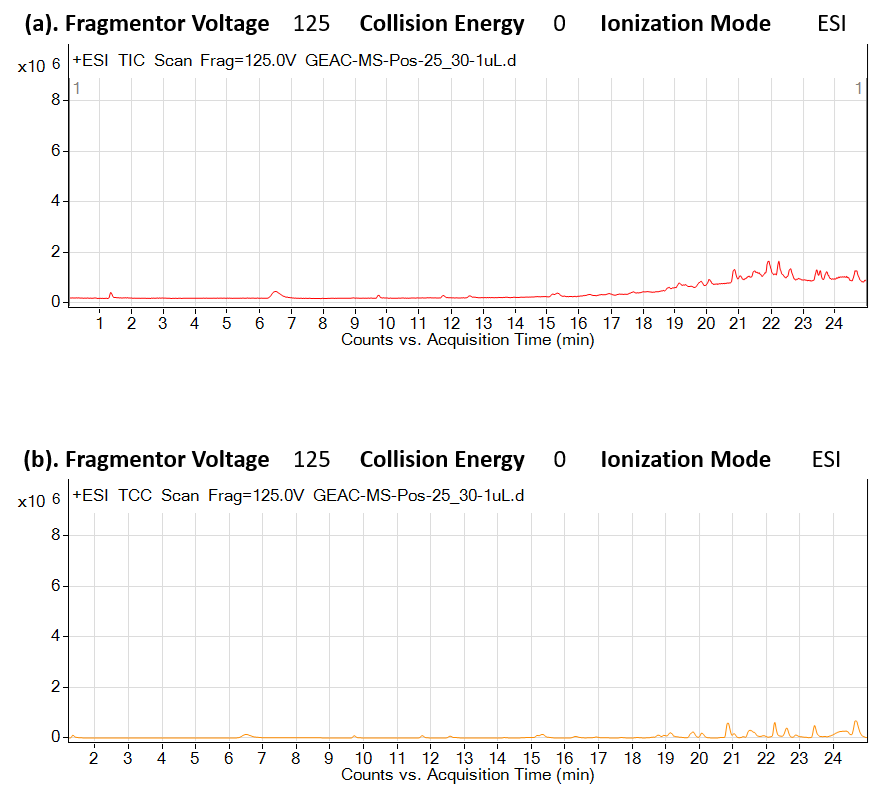
**FIGURE S2.** *G. edulis* ethyl acetate extract was subjected to LC-MS qualitative analysis using positive ion mode. **a)**Total ion current (TIC) chromatogram and **b)** Total compound chromatogram (TCC).

**FIGURE S3.** *G. edulis* acetone extract was subjected to LC-MS qualitative analysis using positive ion mode. **a)**Total ion current (TIC) chromatogram and **b)** Total compound chromatogram (TCC).

**GC-MS analysis of *G. edulis* crude sequential extracts:**

**FIGURE S4.** GC-MS fingerprinting of ethyl acetate sequential extract of *G. edulis*. Chemical determinants were identified on based of % peak area.

**FIGURE S5.** GC-MS fingerprinting of acetonic sequential extract of *G. edulis*. Chemical determinants were identified on based of % peak area.

**GC-MS analysis of *G. edulis* direct extracts**


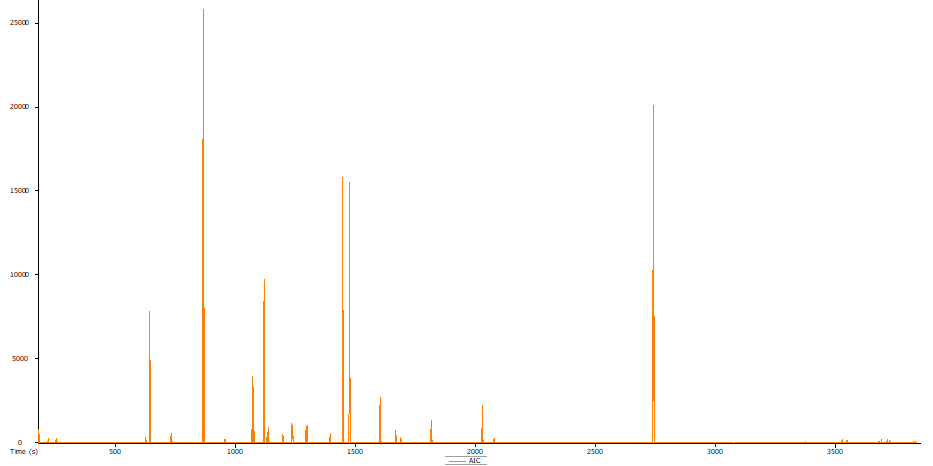


**FIGURE S6.** GC-MS fingerprinting of ethyl acetate extract of *G. edulis*. Chemical determinants were identified on based of % peak area.


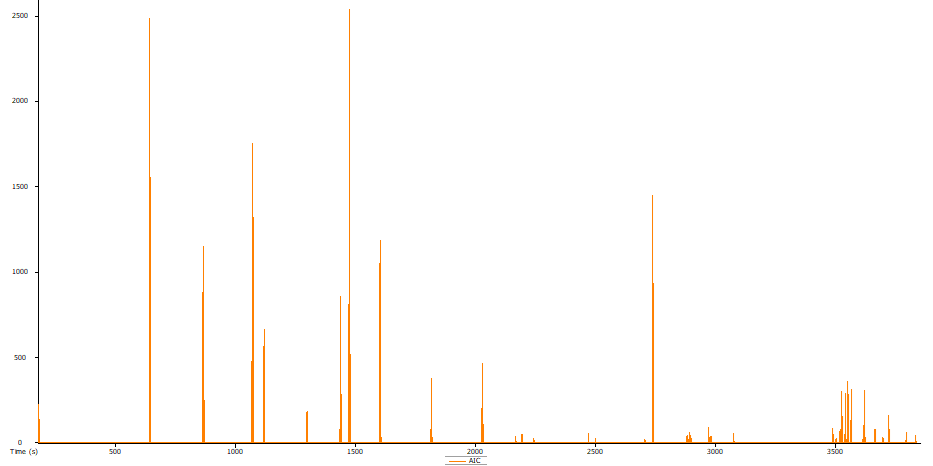


**FIGURE S7.** GC-MS fingerprinting of acetonic sequential direct extract of *G. edulis*. Chemical determinants were identified on based of % peak area.
